# Unravelling Intrinsic and Extrinsic Factors Shaping the Rich Communities on Lizard Skin

**DOI:** 10.1111/1758-2229.70172

**Published:** 2025-08-06

**Authors:** Chava L. Weitzman, Kimberley Day, Karen Gibb, Gregory P. Brown, Angga Rachmansah, Keith Christian

**Affiliations:** ^1^ Research Institute for the Environment and Livelihoods CDU Darwin Australia; ^2^ School of Natural Sciences Macquarie University Sydney Australia

**Keywords:** amplicon sequencing, bacterial stochasticity, cutaneous microbiome, gecko skin

## Abstract

Geckos have high skin bacterial diversity, even though gecko skin has antimicrobial, self‐cleaning properties. To gain a better understanding of environment–animal–microbiome interactions in these reptiles, we investigated skin bacteria on seven northern Australian gecko species from five sites and two seasons (*n* = 234) and found support for our hypotheses of divergent communities between species, sites and seasons. Despite that support, predictor variables had low explanatory power, which increased when focusing within a site or species, explaining up to 40% and 27% of the variation among samples at a site or on a species found in multiple sites, respectively. Weather explained even less variation, as temperature and rainfall did not account for site and season differences. Low explanatory power of these variables indicates that additional factors, or stochasticity, explain much of the bacterial assemblage on geckos. Next, research is needed to determine if these low‐biomass communities represent living symbionts. If so, assessing functional similarities, rather than taxonomic profiling, would clarify if bacterial communities have interactive roles with gecko hosts or represent short‐lived environmental hitch‐hikers and relic DNA.

## Graphical abstract

Geckos host diverse skin bacteria despite low biomass. Geckos in the Australian seasonal tropics hosted divergent skin communities between species, sites and seasons, but with low explanatory power. We suggest assessment of skin bacterial viability before exploring whether functional diversity better explains community patterns than taxonomic diversity in these reptiles.

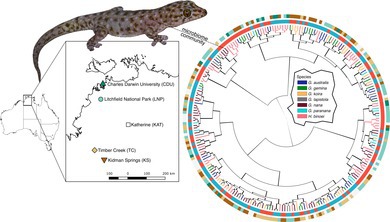

## 1 Introduction

In recent decades, we have gained extraordinary insight into the patterns and roles of bacterial communities in and on animal hosts (symbioses). These discoveries have largely been motivated by a push toward increasing our understanding of the human body (Turnbaugh et al. 2007; Harris‐Tryon and Grice 2022) and a reaction to emerging wildlife diseases (e.g., Walker et al. 2019; Rebollar et al. 2020), leaving many systems understudied. Better awareness of the roles and patterns of bacterial symbionts of wildlife hosts could prepare us to tackle future challenges and threats to wildlife populations proactively, rather than reactively (Ribas et al. 2023).

Terrestrial reptiles have dry, scaly skin that provides a protective layer and site of water loss and heat exchange (Akat et al. 2022). Presumably, the importance of bacteria on terrestrial reptile skin may be similar to that on mammalian or bird skin, contributing to the skin's protective layer against invasion but also interacting with antimicrobial peptides, particularly during wound healing (Harris‐Tryon and Grice 2022; Akat et al. 2022). Previous work has found relatively high bacterial diversity on lizard skin (Weitzman et al. 2018), suggesting less filtering from the external environment and communities more similar to the surrounding environment than those found on amphibians (Walke et al. 2014; Hyde et al. 2016). We know little about the factors that impact these diverse microbial communities on healthy animals, and revealing such patterns could help us to understand the roles of bacteria on reptile skin. This insight would help prepare us to manage future crises such as the emergence of disease or other anthropogenic population declines.

On geckos in particular, data support high levels of richness in skin bacterial communities despite evidence that gecko skin is an unwelcoming site for bacterial colonisation. Gecko skin is covered in spiny micro‐ and nano‐structures that can pierce bacteria (Watson et al. 2015; Li et al. 2016), simultaneously creating an extremely hydrophobic surface that self‐cleans as water drops roll off the animal (Watson et al. 2015; Riedel et al. 2020). As reptiles, geckos regularly shed their skin, which helps to remove ectoparasites (Fushida et al. 2020) and likely also reduces bacterial loads as found in other wildlife (Meyer et al. 2012; Cramp et al. 2014; Weitzman et al. 2025). Consequently, bacteria on geckos face obstacles to their survival, and the morphology and physiology of the skin itself likely play a role in filtering colonising taxa.

We explored bacterial assemblages on geckos in the wet–dry tropics of Northern Territory, Australia to identify the main factors influencing natural patterns on visibly healthy, wild geckos. We collected skin bacterial samples from seven species across five sites in two seasons, encompassing large weather fluctuations and multiple microhabitat types to break down the roles of multiple factors on community patterns. We use these data to describe basic details of gecko skin communities and address four hypotheses. (1) Gecko species, even those closely related, harbour unique skin communities. This is an extension of our previous findings of different communities on other, less closely related lizards, as well as data from others on snakes (Weitzman et al. 2018; Walker et al. 2019). Differing morphology, physiology and ecology among species would contribute to support for this hypothesis. (2) Gecko bacterial communities differ by sampling site, as found in other systems (Avena et al. 2016; Christian et al. 2018; Lavrinienko et al. 2018). This would be influenced by differing bacteria available to colonise at different sites due to differing environments and distance (Martiny et al. 2006). (3) Community similarity will correspond with more similar gecko microhabitats (e.g., ground‐dwelling and those on/around small rocks on the ground versus arboreal), suggesting an influence of the immediate environment on bacterial colonisation and maintenance. (4) Finally, bacterial communities on geckos change with time, specifically seasonally. Temporal skin community changes have been found in snakes (Walker et al. 2019), while a seasonal effect in frogs is apparent and can be associated with other changes such as behaviour (activity, microhabitat use) and disease (Longo et al. 2015; Varela et al. 2018; Douglas et al. 2021). Within this hypothesis, we test the prediction that weather conditions explain gecko skin diversity, suggesting that rainfall and temperature specifically drive bacterial changes, which could be impacted by factors such as bacterial growing conditions and weather‐related host behavioural and physiological changes.

## 2 Methods

### 2.1 Study System, Sites and Weather Data

**FIGURE 1:**
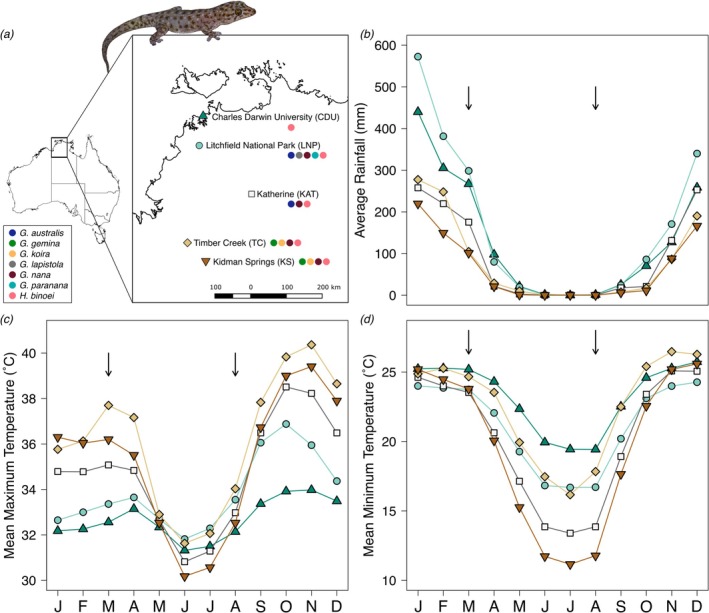
Map and average monthly weather of five sampling sites in the Northern Territory, Australia. (a) Inset map of five sites along a 415 km latitudinal gradient. Coloured circles indicate which gecko species were sampled from each site (*Gehyra* spp. or *Heteronotia binoei* ). (b–d) For context on the climate at the sampling sites, average monthly rainfall and temperatures for 2011–2020 at each site. X‐axis labels represent chronological months, January–December. Weather data were obtained from the Australian Bureau of Meteorology (http://www.bom.gov.au/climate/data/). Arrows indicate seasons when gecko skin bacteria were collected.

Study sites included five locations in the Northern Territory of Australia spanning 415 km along a 3.76° latitudinal gradient (Figure 1). The five sites were chosen to span a range of climates while also allowing for most gecko species to be sampled from more than one location (Table 1). The region is in the wet–dry seasonal tropics, with sites farther from the equator experiencing less rainfall in the wet season and greater temperature fluctuations, with higher maximum temperatures in the wet season and lower minimum temperatures in the dry season (Figure 1). Sampling occurred in the late dry season 2022 (8 August to 26 September) and late wet season 2023 (7 February to 2 April) in hopes of maximising the impacts of seasonal effects.

**TABLE 1:** Skin bacterial community sample sizes used in analyses. Microhabitat type, substrate index and sample sizes per site are shown for each species. Sample sizes include dry/wet values. Asterisks indicate samples included in the sequencing run but removed during normalisation due to low read counts. Sample sizes of species from local sites are provided in Table S2.

Species	Microhabitat	Substrate index	Charles Darwin University	Litchfield National Park	Katherine	Timber Creek	Kidman Springs
*Gehyra australis*	Arboreal	5		7/7	7/9		
*Gehyra gemina*	Arboreal	5				7/7	7/7
*Gehyra koira*	Large rocks/boulders, arboreal	4				6*/7	6*/9
*Gehyra lapistola*	Large rocks/boulders, arboreal	4		5**/7			
*Gehyra nana*	Small rocks, ground	2		7/7	7/7	7/7	7/9
*Gehyra paranana*	Medium rocks	3		7/7			
*Heteronotia binoei*	Ground	1	7/7	7/7	7/8	7/0	7/3

The genus *Gehyra* (Gekkonidae) is speciose in the sampling locations, with some species recently described (Oliver et al. 2020). Among four sampling sites, we sampled six *Gehyra* spp. inhabiting varying microhabitats (Table 1, Figure 1). Two species (*Gehyra lapistola*, *Gehyra paranana*) have small distributions and were only sampled from Litchfield National Park. Three species (*Gehyra australis*, *Gehyra gemina*, *Gehyra koira*) were sampled from two locations each. *Gehyra nana* has a wider distribution and was sampled from four sites. A seventh species, *Heteronotia binoei* (Gekkonidae), is a widespread ground gecko in Australia and was sampled from all five sites.

We collected weather data from the Australian Bureau of Meteorology (http://www.bom.gov.au/climate/data/) from weather stations closest to the sampling sites (Table S1). For 1, 3, 7, 15, 30, 90 and 180 days leading up to each sampling date for each location, we gathered data on average minimum and maximum temperatures and total rainfall. As many of these values correlate, we reduced the weather variables, latitude and longitude with principal components (PC) analysis. The first two PC axes (Figure S1a) explained 75% of the variation, and we used these two axes as explanatory variables in analyses (see *Statistics* below). PC3 (not analysed) accounted for another 8.4% of the variation. PC1 reflects the seasonal differences, particularly long‐term rainfall and minimum temperatures. PC2 reflects the latitudinal gradient, where sites farther south (higher PC2) are drier in the wet season and can get much hotter than sites farther north.

### 2.2 Sampling and Amplicon Sequencing

Geckos were hand‐captured in the evening and kept in individual clean, dry cloth bags until sampling the same night or the following morning (sample sizes in Table 1). Transient bacteria were rinsed off with 75 mL Milli‐Q water before swabbing the gecko with a flocked swab to collect bacteria from the entire body, avoiding the cloaca and head. Swabs were preserved in 300 μL Zymo DNA/RNA Shield (Zymo Research, Irvine, CA, USA) and chilled in the field until frozen at −20°C. DNA was extracted with the DNeasy Blood and Tissue Kit (Qiagen, Valencia, CA, USA) using the Gram‐positive protocol.

DNA was sent to Ramaciotti Centre for Genomics for library prep and amplicon sequencing, targeting a portion of the bacterial V4 region of 16S rRNA with the Earth Microbiome Project 515F/806R primers (Caporaso et al. 2011; Apprill et al. 2015; Parada et al. 2016) and 2 × 250 bp chemistry on an Illumina MiSeq. The sequencing run included 10 control swab samples exposed to the air, water and gloves during sampling and extracted alongside gecko samples, as well as additional samples collected for a separate gecko experiment. With DADA2 in QIIME2 v2023.7 (Callahan et al. 2016; Bolyen et al. 2019), paired‐end reads were denoised, including primer removal and truncating (218 forward, 166 reverse) bases to determine amplicon sequence variants (ASVs). Merged reads were assigned taxonomy with sklearn using a 515F/806R‐specific classifier based on the Silva v138 database (Pedregosa et al. 2011; Quast et al. 2012; Yilmaz et al. 2014). We kept bacterial ASVs, further removing those identified as chloroplast and mitochondria. Decontam with the default threshold of 0.1 removed contaminant sequences based on the 10 control samples. Because DADA2 may retain spurious ASVs (Reitmeier et al. 2021), those never reaching at least 0.1% abundance in a sample, and those only present in one sample, were removed.

Based on rarefaction curves, analyses were run on data normalised to 6557 reads per sample, removing four samples with the lowest reads (Table 1). Representative sequences were aligned in QIIME2 with mafft and mask with default parameters (Lane 1991; Katoh and Standley 2013). A phylogeny was produced with raxml‐rapid‐bootstrap with a seed of 1723, rapid bootstrap seed of 9384 and GTRCAT substitution model, which was then midpoint rooted for UniFrac calculations (Stamatakis et al. 2008; Stamatakis 2014). Diversity metrics of interest included ASV richness and Pielou's evenness to represent alpha diversity, and unweighted and weighted UniFrac as beta diversity.

### 2.3 Statistics

We first assessed large‐scale community patterns in beta diversity with permutational multivariate ANOVAs (PERMANOVAs) with adonis in QIIME2. For each metric, we conducted two analyses, the first to determine effects of site, species and season on diversity, and the second to measure effects of the two environmental PCs, with host species as a covariate. We used post hoc pairwise PERMANOVAs to further discern site and species differences. Because our sampling regime was not fully factorial, we then subdivided the dataset by site to analyse the predictors of species and season, and separately subdivided by species to analyse the predictors of site and season.

We analysed alpha diversity metrics in R v4.3.1 (R Core Team 2023) with ANOVAs where appropriate. Within each sampling site, we assessed the significance of host species, season and their interaction on richness. As only one species was sampled from Charles Darwin University (CDU), those data were analysed based on season alone. Similarly, for each species, we analysed the impacts of site (where relevant), season and their interaction (where relevant). Some groups of data, particularly in the evenness metric, violated ANOVA assumptions of normality and equal variance, so they were analysed with Kruskal–Wallis tests. Post hoc pairwise contrasts were calculated with emmeans (Lenth 2024) or Dunn's tests (Ogle et al. 2023). For each species sampled from more than one location, we further assessed the impacts of environmental PC1 and PC2 on microbial richness with linear models (lm) in R.

Beta diversity was analysed in a similar manner (within each site and within each season) with adonis in QIIME2, further assessing post hoc pairwise PERMANOVAs and analysis of beta dispersion with permdisp where relevant. First, for each sampling site, we assessed the impacts of species (excluding at CDU where only one species was sampled) and site on bacterial community composition. We included local sampling site as a covariate in beta diversity analyses for Litchfield National Park, Timber Creek and Kidman Springs, as samples were collected across a large area (Table S2). Second, for each species, we assessed the impacts of site and season on beta diversity. Third, for each species sampled from multiple sites, we also analysed the effects of environmental PC1 and PC2 in a separate adonis analysis to determine the impact of climate and weather conditions.

Finally, we separately analysed weighted UniFrac of all samples from each season with adonis to address our third hypothesis regarding the influence of microhabitat on bacterial composition. We assigned a metric of substrate type to each species sampled (Table 1), increasing in value from the ground up, from ground‐dwelling (index = 1) through tree‐dwelling (index = 5). The impact of microhabitat substrate type was analysed after accounting for site.

Alongside statistical analyses, we determined core ASVs (present in 80% of samples) within each species and within each site as another metric of similarities among groups of samples. Core genera were also detected for each gecko species. We used hierarchical clustering of weighted UniFrac distances using Ward's method (Murtagh and Legendre 2014) and nonmetric multidimensional scaling (NMDS) with the vegan and phyloseq packages in R v4.3.1 (McMurdie and Holmes 2013; R Core Team 2023; Oksanen et al. 2024) to visualise large‐scale patterns of community composition in our samples and provide additional visualisations of principal coordinates analyses in the supplemental materials.

## 3 Results

**FIGURE 2:**
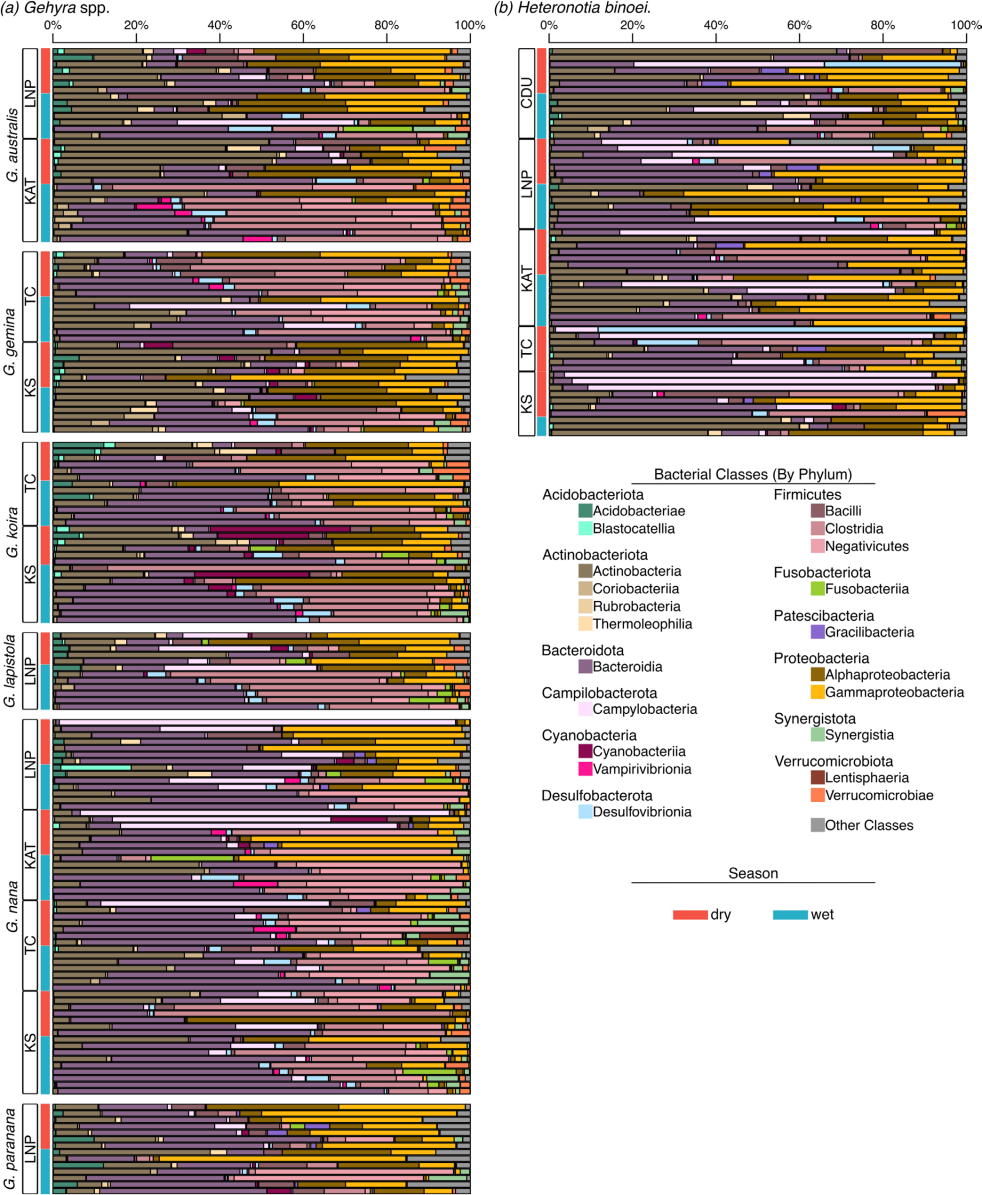
Barplots of bacterial classes present on the skin of geckos, split by host species: (a) Six *Gehyra* spp. and (b) *Heteronotia binoei* . Within each species, data are separated by sampling site and season. Only classes representing at least 5% of the relative abundance in any one sample are included, with other classes and unclassified bacteria grouped into ‘other classes’. CDU, Charles Darwin University; KAT, Katherine; KS, Kidman Springs; LNP, Litchfield National Park; TC, Timber Creek.

Filtered gecko data (*n* = 234 samples) contained 6,829,706 sequences in 5507 ASVs, with 2935–79,096 sequences per gecko sample. Richness in the normalised data ranged from 25 to 451 ASVs. Bacterial communities on geckos were largely comprised of the phyla Bacteroidota (predominantly Bacteroidia, Figure 2), Firmicutes (Clostridia), Proteobacteria (Alphaproteobacteria and Gammaproteobacteria) and Actinobacteriota (Actinobacteria). Two classes comprised over half of the normalised reads on many individuals: Bacteroidia was abundant on 30 geckos, mostly *G. nana* (*n* = 12), *H. binoei* (*n* = 8) and *G. koira* (*n* = 6); Campilobacterota, specifically *Helicobacter*, comprised over half of the relative abundance on 14 geckos, also mostly ground‐ and low rock‐dwelling *G. nana* (*n* = 5) and *H. binoei* (*n* = 8).

**FIGURE 3:**
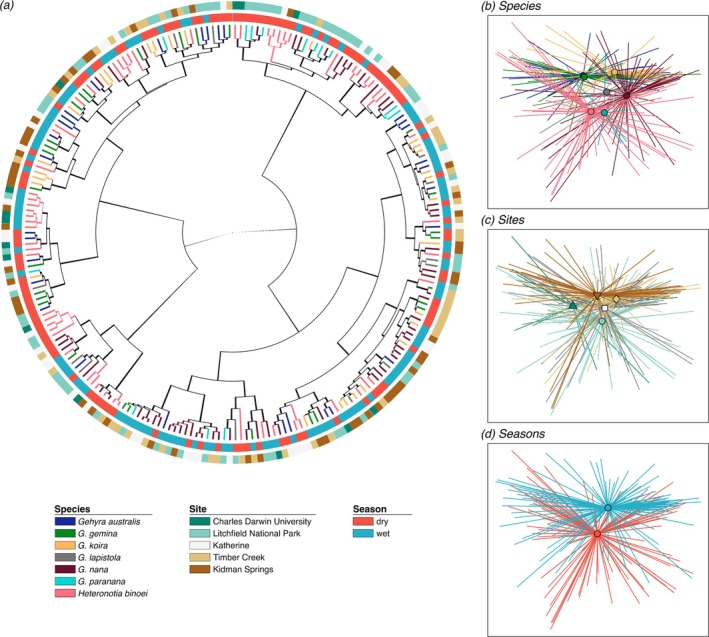
Gecko skin bacterial communities based on weighted UniFrac distances. (a) Dendrogram made using hierarchical clustering with Ward's method. Tips colour‐coded by species. Inner ring is coloured by season. Outer ring is coloured by sampling site. (b–d) Ordinations are nonmetric multidimensional scaling (NMDS), visualising two axes, stress = 0.18. Ordinations colour‐code the samples and their averages by (b) host species, (c) site, and (d) season.

In the full dataset, site, species and season were all significant predictors of beta diversity, despite no strong patterns visible among these variables (Figure 3; unweighted UniFrac: site *F*_(4,218)_ = 3.47, *R* ^2^ = 0.053, *p* = 0.001; species *F*_(6,218)_ = 3.31, *R* ^2^ = 0.076, *p* = 0.001; season *F*_(1,218)_ = 8.96, *R* ^2^ = 0.034, *p* = 0.001; weighted UniFrac: site *F*_(4,218)_ = 3.21, *R* ^2^ = 0.049, *p* = 0.001; species *F*_(6,218)_ = 4.20, *R* ^2^ = 0.095, *p* = 0.001; season *F*_(1,218)_ = 8.48, *R* ^2^ = 0.032, *p* = 0.001). In pairwise comparisons of weighted UniFrac, *G. lapistola* was only significantly different from *G. paranana*, the other species sampled only from Litchfield National Park. Communities on *G. australis* , an arboreal gecko, were similar to those of two other arboreal species (*G. gemina*, *G. koira* ) sampled from different sites than *G. australis* . In contrast, most species differed in unweighted UniFrac (except *G. lapistola* compared with *G. australis* and *G. koira* ). Among the sites, all localities differed in unweighted UniFrac; notable results in weighted UniFrac were that geckos from Timber Creek and Kidman Springs, the two sites furthest south, had similar bacterial communities to each other, and communities from Katherine were similar to all other sites. Environmental PC1 and PC2 were significant predictors of one or both beta diversity metrics, but explained less than 3% of the variation in either (unweighted UniFrac: PC1 *F*_(1,221)_ = 6.71, *R* ^2^ = 0.027, *p* = 0.001; PC2 *F*_(1,221)_ = 1.20, *R* ^2^ = 0.008, *p* = 0.008; weighted UniFrac: PC1 *F*_(1,221)_ = 6.17, *R* ^2^ = 0.024, *p* = 0.001; PC2 *F*_(1,221)_ = 1.10, *R* ^2^ = 0.004, *p* = 0.3).

### 3.1 Species Differences

**FIGURE 4:**
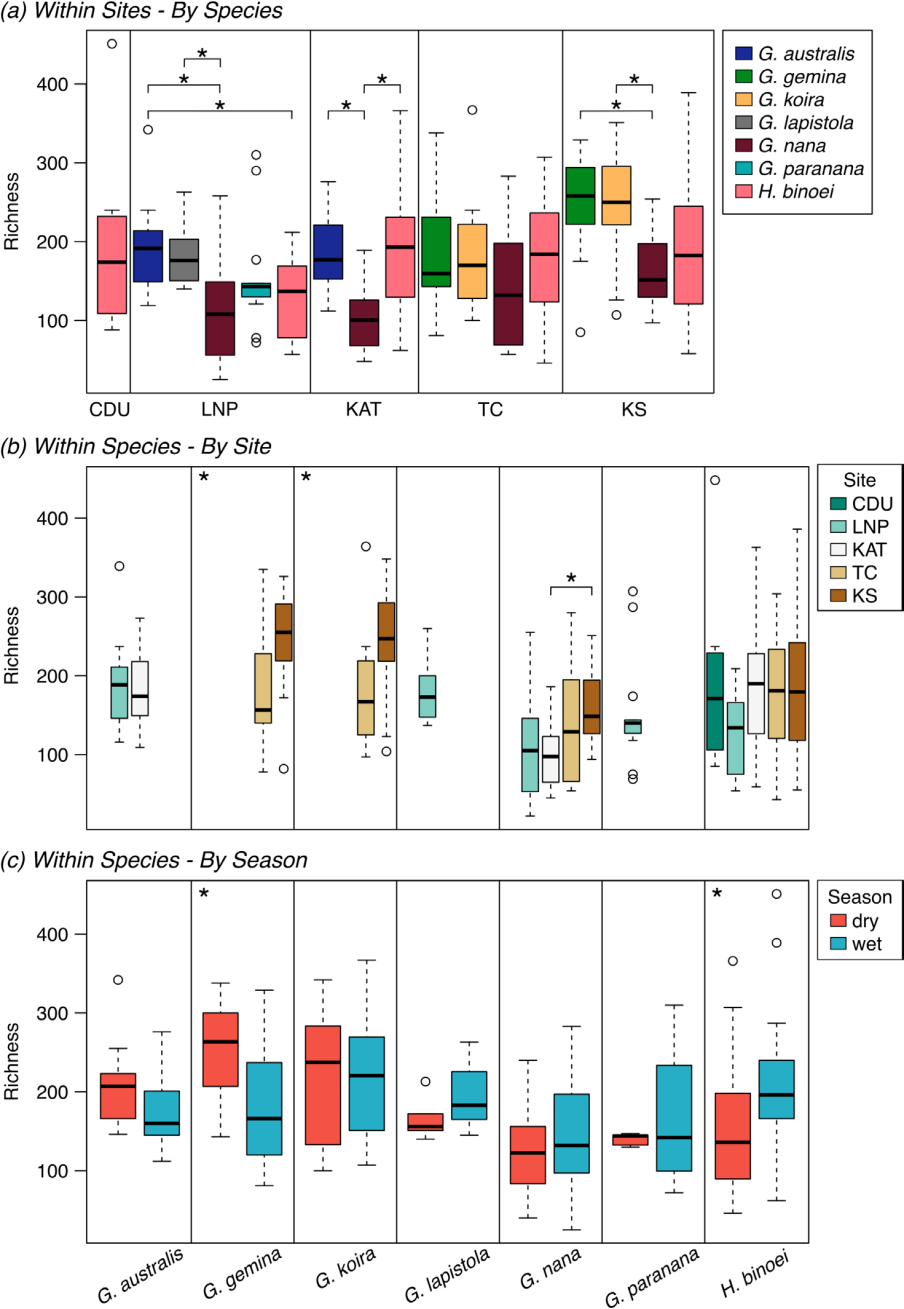
ASV richness in skin bacterial communities on *Gehyra* spp. and *Heteronotia binoei* geckos among (a) species per site, (b) sites per species and (c) season per species. Sites arranged north to south, corresponding with decreasing annual precipitation. Asterisks denote significant differences. CDU, Charles Darwin University; KAT, Katherine; KS, Kidman Springs; LNP, Litchfield National Park; TC, Timber Creek.

Gecko species differed in skin bacterial richness, evenness, community membership and composition at most sites (Table 2, Figure 4), explaining up to 15% of beta diversity variation within a site. At three sites, *G. nana* had lower richness on its skin than other species (Figure 4a), while *H. binoei* as well as *G. nana* often had reduced evenness (Figure S4a). Ordinations of beta diversity did not show strong clustering of samples by species (Figures 3, S6–S9), though most pairwise comparisons between species in beta diversity were significant (Tables S5–S7). Inclusion of local sampling site as a covariate may have masked some impact of species, for instance where one species was completely sampled from a local site separate from the others ( *G. australis* at Litchfield National Park, *H. binoei* at Timber Creek, *G. koira* at Kidman Springs; Table S2). Species at Litchfield National Park and Kidman Springs differed in beta dispersion (Table S9).

**TABLE 2:** Results of analyses of four diversity metrics by site. Local site was included as a covariate for analyses of beta diversity of most sites. **Bold** denotes significance.

Predictor(s)	Richness		*Pielou's evenness*		*Unweighted UniFrac*		*Weighted UniFrac*	
	*F* (df)	*p*	*χ* ^2^ (df)	*p*	*R* ^2^	*p*	*R* ^2^	*p*
Charles Darwin University								
Season	4.309 (1,12)	0.06	**3.92 (1)**	**0.048**	**0.139**	**0.002**	0.150	0.09
(Residuals)					0.861		0.850	
Litchfield National Park								
Local site					**0.078**	**0.001**	**0.085**	**0.003**
Species	4.922 (4,58)	**0.002**	**12.30 (4)**	**0.02**	**0.091**	**0.001**	**0.107**	**0.003**
Season	2.309 (1,58)	0.1	0.0009 (1)	1	**0.047**	**0.001**	**0.051**	**0.001**
Spp × Season	2.454 (4,58)	0.06			**0.068**	**0.03**	**0.078**	**0.04**
(Residuals)					0.716		0.679	
Katherine								
Species	9.084 (2,39)	**0.0006**	**22.66 (2)**	**< 0.0001**	**0.123**	**0.001**	**0.113**	**0.002**
Season	0.016 (1,39)	0.9	0.32 (1)	0.6	**0.089**	**0.001**	**0.057**	**0.009**
Spp × Season	0.726 (2,39)	0.5			**0.067**	**0.02**	**0.119**	**0.001**
(Residuals)					0.721		0.710	
Timber Creek								
Local site					**0.141**	**0.001**	**0.128**	**0.05**
Species	0.839 (3,41)	0.5	3.97 (3)	0.3	0.076	0.07	0.088	0.1
Season	1.352 (1,41)	0.3	2.09 (1)	0.1	0.026	0.1	0.013	0.7
Spp × Season	2.141 (2,41)	0.1			0.036	0.6	0.036	0.5
(Residuals)					0.721		0.736	
Kidman Springs								
Local site					**0.089**	**0.001**	**0.089**	**0.006**
Species	7.359 (3,47)	**0.0004**	**17.01 (3)**	**0.0007**	**0.103**	**0.001**	**0.149**	**0.001**
Season	0.868 (1,47)	0.4	**3.95 (1)**	**0.047**	**0.045**	**0.001**	0.035	**0.03**
Spp × Season	4.895 (3,47)	**0.005**			**0.068**	**0.01**	**0.127**	**0.004**
(Residuals)					0.695		0.598	

The five gecko species sampled at more than one site had zero ( *G. koira* , *G. nana* ) to three (*G. gemina*) core ASVs, which were relatively rare with median relative abundances only up to 0.8% (Table S3). At the genus level, most host species had many more core taxa, which were still relatively rare in the bacterial communities (Table S4). The two gecko species that were only sampled from Litchfield National Park (*G. lapistola*, *G. paranana*) had larger core bacterial communities than the others and greater overlap in their core taxa (Figure S2), unsurprising considering their lower sample sizes and reduced spatial variation of samples.

### 3.2 Site Differences

In four of the five gecko species sampled from multiple sites, communities varied by site in at least one diversity metric (Table 3, Figure 4b), explaining up to 16% of variation in beta diversity, with minimal clustering in hierarchical clustering and ordinations (Figures 3, S10–S13, S16). In *G. gemina* and *G. koira* , richness at Kidman Springs was higher than that at nearby Timber Creek (Figure 4), with evenness also higher at Kidman Springs in *G. gemina* (Figure S4b). Most site‐level pairwise comparisons were not significant, except for some unweighted UniFrac comparisons in *G. nana* (Table S8). We found no differences in beta dispersion between sites for a species (Table S10). Sites where multiple gecko species were sampled had few core ASVs, each with median relative abundance < 2% (Table S3).

**TABLE 3:** Results of analyses of four diversity metrics by species. Test statistic for analyses of categorical variables with alpha diversity is *F* value from ANOVA for most. Asterisk denotes Kruskal–Wallis chi‐squared values. Test statistic for linear models of environmental PC1 and PC2 on richness are t values. **Bold** denotes significance.

Predictor(s)	*Richness*		*Pielou's evenness*		*Unweighted UniFrac*		*Weighted UniFrac*	
	Test statistic (df)	*P*	Test statistic (df)	*P*	*R* ^*2*^	*P*	*R* ^*2*^	*P*
*Gehyra australis*								
Site	0.070 (1,26)	0.8	0.842 (1,26)	0.4	0.052	0.053	0.066	0.07
**Season**	3.652 (1,26)	0.07	**6.216 (1,26)**	**0.02**	**0.116**	**0.001**	**0.135**	**0.006**
Site × season	2.026 (1,26)	0.2	0.497 (1,26)	0.5	0.052	0.06	0.064	0.07
(Residuals)					0.779		0.735	
**Env PC1**	−2.49	**0.02**	**−2.56**	**0.03**	**0.062**	**0.04**	0.043	0.2
**Env PC2**	1.30	0.2	**2.47**	**0.02**	0.049	0.1	0.045	0.2
(Residuals)					0.889		0.912	
*Gehyra gemina*								
**Site**	7.681 (1,24)	**0.01**	3.90 (1)*	**0.048**	**0.097**	**0.002**	**0.160**	**0.001**
**Season**	7.984 (1,24)	**0.009**	2.44 (1)*	0.1	**0.076**	**0.007**	0.060	0.1
Site × season	1.456 (1,24)	0.2			0.046	0.1	0.045	0.2
(Residuals)					0.781		0.734	
**Env PC1**	−3.19	**0.004**	2.04	0.05	**0.093**	**0.003**	**0.083**	**0.03**
**Env PC2**	−3.68	**0.001**	**−2.48**	**0.02**	**0.071**	**0.01**	**0.117**	**0.01**
(Residuals)					0.836		0.801	
*Gehyra koira*								
**Site**	6.124 (1,24)	**0.02**	0.5 (1,26)	0.5	**0.069**	**0.02**	0.039	0.3
**Season**	0.050 (1,24)	0.8	1.81 (1)*	0.2	**0.081**	**0.005**	**0.109**	**0.03**
Site × season	0.447 (1,24)	0.5			0.034	0.4	0.030	0.4
(Residuals)					0.816		0.822	
**Env PC1**	−0.67	0.5	−1.21	0.2	**0.081**	**0.01**	**0.112**	**0.02**
**Env PC2**	−1.00	0.3	−1.34	0.2	**0.065**	**0.03**	0.037	0.3
(Residuals)					0.854		0.851	
*Gehyra lapistola*								
**Season**	1.528 (1,10)	0.2	2.901 (1,10)	0.1	**0.225**	**0.004**	**0.362**	**0.009**
(Residuals)					0.775		0.638	
*Gehyra paranana*								
**Season**	0.004 (1)*	0.9	0.016 (1,12)	0.9	**0.118**	**0.02**	**0.189**	**0.01**
(Residuals)					0.882		0.811	
*Gehyra nana*								
**Site**	3.507 (3,50)	**0.02**	6.63 (3)*	0.08	**0.111**	**0.001**	**0.089**	**0.02**
**Season**	1.261 (1,50)	0.3	3.26 (1)*	0.07	**0.067**	**0.001**	**0.071**	**0.002**
**Site** × **season**	1.027 (3,50)	0.4			**0.063**	**0.050**	0.067	0.1
(Residuals)					0.760		0.773	
**Env PC1**	1.20	0.2	1.46	0.2	**0.052**	**0.002**	0.047	0.02
Env PC2	0.43	0.7	0.05	1	0.026	0.08	0.010	0.7
(Residuals)					0.922		0.943	
*Heteronotia binoei*								
**Site**	1.416 (4,51)	0.2	7.60 (4)*	0.1	**0.094**	**0.005**	0.080	0.1
**Season**	11.593 (1,51)	**0.001**	6.46 (1)*	**0.01**	**0.045**	**0.001**	**0.096**	**0.001**
Site × season	1.257 (3,51)	0.3			0.049	0.4	0.048	0.4
(Residuals)					0.813		0.775	
**Env PC1**	2.53	**0.01**	**2.54**	**0.01**	**0.035**	**0.008**	**0.067**	**0.001**
**Env PC2**	0.07	0.9	−1.87	0.07	**0.026**	**0.04**	0.009	0.7
(Residuals)					0.940		0.924	

### 3.3 Microhabitat Patterns

**FIGURE 5:**
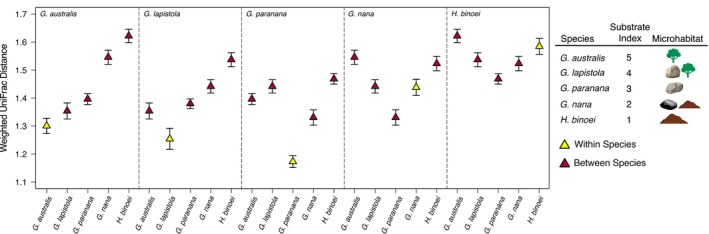
Average pairwise weighted UniFrac distances within and between species sampled at Litchfield National Park. The five species are ordered based on microhabitat from arboreal to terrestrial. Error bars denote standard error.

Through qualitative examination of pairwise weighted UniFrac distances, we expected species in more similar microhabitats to have more similar communities (lower distances). Five species from Litchfield National Park inhabit a range of microhabitat substrates. In the arboreal/semi‐arboreal *G. australis* and *G. lapistola*, bacterial communities were more similar to each other and to those on large rocks (*G. lapistola* vs. *G. paranana*) compared to those geckos on the ground and low‐lying rocks (Figure 5). The pattern of lower distances between more similar microhabitats disappeared for the gecko species lower to the ground.

We found a greater influence of microhabitat substrate (i.e., explaining a larger proportion of variation) in the dry season compared to the wet season (dry: *F* = 14.62, *R* ^2^ = 0.108, *p* = 0.001; wet: *F* = 2.48, *R* ^2^ = 0.021, *p* = 0.051).

### 3.4 Weather and Environment

We assessed the influence of weather in two ways, first using season (dry vs. wet) as a categorical predictor variable, then analysing environmental PC1 and PC2 as continuous predictors.

In alpha diversity, there were differences between the sampling seasons for a few species (richness in *G. gemina* and *H. binoei* ; evenness in *G. australis* and *H. binoei* ), but within each metric, the bacterial communities did not change in the same direction between dry and wet seasons (Table 3, Figures 4c, S4c). At Kidman Springs, gecko species formed two groups of similar richness in the dry but not the wet season due to an increase in richness in *H. binoei* (Figure S3). At this site, only *H. binoei* had differing richness between the two seasons. Beta diversity differed between the seasons in most analyses, accounting for up to 14% of variation in a site (unweighted UniFrac at CDU) and 36% of variation in a species (weighted UniFrac for *G. lapistola*; Tables 2 and 3, Figures S5–S16). The interaction between season and either site ( *G. nana* , Table S11) or species was a frequent predictor of beta diversity. In samples from the Katherine site and those collected from *G. nana* among the sites, wet season samples were more similar than dry season samples (differing beta dispersion; Tables S9, S10). This contrasts with *G. paranana* samples, which were more similar in the dry season than the wet.

PC1 (corresponding with minimum temperature, long‐term rain and long‐term maximum temperature) significantly predicted bacterial richness on *G. australis* , *G. gemina* and *H. binoei* , but not in the same direction throughout (Table 3, Figure S1). PC2 (maximum temperature and rainfall, both corresponding with latitude) only correlated with bacterial richness on *G. gemina*, with declines in richness as PC2 increased. For evenness, we similarly found different directional relationships among species; PC1 positively correlated with evenness in *H. binoei* but negatively correlated with evenness in *G. australis* , while increasing PC2 corresponded with reduced evenness in *G. gemina* but increased evenness in *G. australis* (Figure S1). On species sampled from multiple sites, environmental variables (especially PC1—minimum temperatures) explained some of the differences in beta diversity in our samples but accounted for less variation than the categorical variables (up to 20% variation of weighted UniFrac for *G. gemina*).

## 4 Discussion

The aim of our analysis of gecko skin bacteria across a 415 km latitudinal and weather gradient was to identify drivers of skin bacterial communities on lizards, an understudied system in microbial ecology. The gecko skin communities sampled had high diversity and low similarity among samples, with large overlaps in community structure between species, sites and seasons. Seasonal changes were also not consistent among species. In the effort to discern the relative influences of host and environment on host microbiomes (Kohl 2020), we found mixed, weak support of our four hypotheses, with explanatory power less than 20% for the combinations of predictors of interest for the full dataset. By focusing on patterns within sites and species, we found improved evidence of community divergence among host gecko species, site and season, with up to 40% of the variance in a group of geckos explained by our predictor variables. The low explanatory power of species for bacterial communities on geckos is less than that found in other studies of less‐related lizards and snakes, where species explained 28% (Weitzman et al. 2018) to 45% (Walker et al. 2019) of variance in skin bacterial communities. For comparison, sympatric frogs (55%; Weitzman et al. 2018) and sympatric amphibians (frogs and salamander, 87%; McKenzie et al. 2012) have greater community divergence between species than what we find on terrestrial squamate reptiles. Geckos harbouring few and rare core taxa further supports minimal bacterial overlap within species across space and time. Regardless, gecko species did have significantly differing communities, which may be impacted by intrinsic factors as well as microhabitat. As these geckos tend to occupy distinct microhabitats, causes of species‐level differences cannot be determined with this dataset.

In the dry season in particular, gecko microhabitat was a relatively strong predictor of bacterial communities, which, alongside the significance of weather, supports seasonal community differences. The influence of microhabitat, as well as reduced richness and greater dispersion in the dry season (found in some of the gecko species), could be a result of reduced gecko activity reducing exposure to environmental bacterial reservoirs. Although we do not have seasonal activity data for the species in this study, many lizards in the wet‐dry tropics of northern Australia are significantly less active in the dry season compared to the wet season. This has been quantified both directly (by telemetry) and indirectly through seasonal energy balance analyses for one species of gecko (Christian et al. 1998) and multiple other lizard taxa (Christian et al. 1995, 1996, 2003; Smith et al. 2008).

Presumably, weather may impact gecko skin communities by influencing growing conditions, while rain also cleans microbes off of skin (Watson et al. 2015; Riedel et al. 2020). Though we predicted that weather itself influences the communities on gecko skin, we found that environmental PCs, which correlate with site and season, were weaker predictors than site and season themselves. Furthermore, the highest explanatory power of season was for groups of geckos with low sample sizes (*G. lapistola* at Litchfield National Park, *H. binoei* at CDU). Alternatively, both *H. binoei* and *G. nana* were sampled from multiple sites where they encountered a range of weather patterns, but despite this, environmental PCs were not strong predictors of microbial patterns. Consequently, other seasonal factors such as available bacteria, exposure to microhabitats (behavioural shifts), microclimate, dietary changes or seasonal physiological variation may shape these communities.

If bacteria on gecko skin have a function interacting with their host, they may assemble based on functional diversity, as many diverse taxa can perform the same functions (Louca et al. 2018). Consequently, studies find stronger patterns of functional groups than taxonomic groups across environmental conditions (e.g., in the ocean; Louca, Parfrey, et al. 2016), as well as highly conserved functional diversity despite high taxonomic variation among individuals of a host species (e.g., bromeliads; Louca, Jacques, et al. 2016). Further studies using metagenomics and metatranscriptomics would provide a better understanding of the functional diversity and whether species, spatial and seasonal patterns arise on widespread geckos.

Though we have repeatedly found bacteria on gecko skin, geckos have skin surfaces unwelcoming to bacterial retention, survival or growth (Watson et al. 2015; Li et al. 2016), and we have no data regarding whether DNA from swab samples represents living taxa. We did not collect samples and morphological data in such a way as to estimate bacterial density on the skin, but quantitative PCR data suggest that bacterial biomass in gecko skin swab samples is low (unpublished data).

As we were unable to explain most of the variance among these low biomass samples, we predict that geckos do not have strong direct interactions with their skin bacteria. Natural variation of bacteria across environments and distance could explain the significant patterns found among our samples (Martiny et al. 2006). In other species, neutral processes have created high diversity in host‐associated microbiomes (e.g., juvenile zebrafish guts; Burns et al. 2016). Quantifying the impact of neutral processes in this system was beyond the scope of this study, necessitating further environmental sampling, including sampling from their daytime refugia where they spend most of their time, but the locations of which were not known. Furthermore, we hypothesise that a large portion of gecko skin bacteria may represent transient colonisation rather than long‐term symbiotic interactions, as geckos acquire skin bacteria from their habitat, which then die in their interaction with the skin surface or are washed away in the wet season rain. Relic DNA from dead bacteria can account for a large proportion of microbiome samples (Lennon et al. 2018), and its presence in low‐biomass samples may have strong impacts on observed diversity. While there are challenges to identifying microbiome‐free hosts and surfaces, many invertebrate animals, as well as certain vertebrate organs, are known to harbour minimal microbes or no symbiotic bacteria (Hammer et al. 2019). Could gecko skin be among this list and naturally bacteria‐free? Alongside a need for further insight into the viability of gecko skin taxa, investigation of the functional redundancy of bacteria among taxonomically diverse communities would clarify whether they play an important role in host health. Alternatively, gecko skin bacteria may largely be hitch‐hikers, impacted by stochastic processes and individual host movements.

## Author Contributions

**Chava L. Weitzman:** conceptualization, data curation, formal analysis, investigation, visualization, writing – original draft, writing – review and editing. **Kimberley Day:** conceptualization, investigation, writing – review and editing. **Karen Gibb:** conceptualization, funding acquisition, resources, writing – review and editing. **Gregory P. Brown:** funding acquisition, writing – review and editing. **Angga Rachmansah:** investigation, writing – review and editing. **Keith Christian:** conceptualization, funding acquisition, investigation, resources, supervision, writing – review and editing.

## Conflicts of Interest

The authors declare no conflicts of interest.

## Supporting information


**Table S1:** Five sampling sites in the Northern Territory, Australia and their weather station data. Distance ranges are included for the three sites with local subsites.
**Table S2:** Sample sizes from local subsites per sampling location. At Litchfield National Park, Tolmer Falls is 13+ km from the other local sites, which are within 2.75 km of each other. At Timber Creek, all local sites are within 2.8 km of each other. At Kidman Springs, the quarry site is approx. 6.25 km from the dump, which is 1 km from the station. Samples from Charles Darwin University and Katherine are excluded here, because at each, the samples were collected within ~0.5 km of each other.
**Table S3:** Core ASVs on the skin of groups of geckos (species or site) at 80% prevalence cut‐off. Asterisks at the beginning of ASV codes indicate ASVs present in the cores of multiple groups.
**Table S4:** Core genera on the skin of gecko species at 80% prevalence cut‐off.
**Table S5:** Results of pairwise PERMANOVA by species for samples from Litchfield National Park.
**Table S6:** Results of pairwise PERMANOVA by species for samples from Katherine.
**Table S7:** Results of pairwise PERMANOVA by species for samples from Kidman Springs.
**Table S8:** Results of pairwise PERMANOVA by site for samples from *Gehyra nana* .
**Table S9:** Results of analyses of beta dispersion with permdisp by sampling site. Each variable was analyzed separately. Differences in beta dispersion were only assessed for variables significant in PERMANOVA. Bold denotes significance.
**Table S10:** Results of analyses of beta dispersion with permdisp by host species (*Gehyra* spp. and *Heteronotia binoei*). Each variable was analysed separately. Differences in beta dispersion were only assessed for variables significant in PERMANOVA. Bold denotes significance.
**Table S11:** *p* values from pairwise PERMANOVA for unweighted UniFrac in *Gehyra nana* skin bacteria.
**Figure S1:** Multiple environmental variables were collected for each sampling location and date, representing recent and long‐term (up to 180 days) weather experienced by the host geckos and their skin microbiomes. These environmental variables were collapsed using a principal components analysis, for which the first two axes (a) accounted for 75% of the variation. Environmental PC1 and PC2 were analysed as predictor variables for richness and evenness on each gecko species sampled from more than one location. (b–f) Raw values and confidence intervals of the relationships between bacterial richness and PC1 (left panels) or PC2 (right panels) for each gecko species. (g–k) Raw values and confidence intervals of the relationships between Pielou's evenness and PC1 (left panels) or PC2 (right panels) for each gecko species. Asterisks denote significant associations.
**Figure S2:** UpSet plot depicting unique and overlapping core genera in *Gehyra* spp. and *Heteronotia binoei*. Warmer colors identify genera in core communities shared by more species.
**Figure S3:** Bacterial richness among the gecko species in the two seasons at the Kidman Springs site, the only site which had a significant interaction between species and season. Letters above boxplots indicate post hoc differences in richness between species within each season separately to visualise the many differences in the dry season not found in the wet. Post hoc tests detected greater richness on *H. binoei* in the wet season versus the dry season.
**Figure S4:** Pielou's evenness in skin bacterial communities on *Gehyra* spp. and *Heteronotia binoei* geckos among (a) species per site, (b) sites per species and (c) season per species. Sites arranged north to south, corresponding with decreasing annual precipitation. Asterisks denote significant differences.
**Figure S5:** Principal coordinates analysis with 95% confidence ellipses of samples collected from geckos (*Heteronotia binoei*) at Charles Darwin University. Point shape and line type denote season.
**Figure S6:** Principal coordinates analysis with 95% confidence ellipses of samples collected from geckos at Litchfield National Park. Color denote species. Point shape and line type (bottom panels) denote season.
**Figure S7:** Principal coordinates analysis with 95% confidence ellipses of samples collected from geckos at Katherine. Colors denote species. Point shape and line type (bottom panels) denote season.
**Figure S8:** Principal coordinates analysis with 95% confidence ellipses of samples collected from geckos at Timber Creek. Colors denote species. Point shape and line type (bottom panels) denote season.
**Figure S9:** Principal coordinates analysis with 95% confidence ellipses of samples collected from geckos at Kidman Springs. Colors denote species. Point shape and line type (bottom panels) denote season.
**Figure S10:** Principal coordinates analysis with 95% confidence ellipses of samples collected from *Gehyra australis*. Colors denote sampling site. Point shape and line type (bottom panels) denote season.
**Figure S11:** Principal coordinates analysis with 95% confidence ellipses of samples collected from *Gehyra gemina*. Colors denote sampling site. Point shape and line type (bottom panels) denote season.
**Figure S12:** Principal coordinates analysis with 95% confidence ellipses of samples collected from *Gehyra koira*. Colors denote sampling site. Point shape and line type (bottom panels) denote season.
**Figure S13:** Principal coordinates analysis with 95% confidence ellipses of samples collected from *Gehyra nana*. Colors denote sampling site. Point shape and line type (bottom panels) denote season.
**Figure S14:** Principal coordinates analysis with 95% confidence ellipses of samples collected from *Gehyra lapistola* at Litchfield National Park. Point shape and line type denote season.
**Figure S15:** Principal coordinates analysis with 95% confidence ellipses of samples collected from *Gehyra paranana* at Litchfield National Park. Point shape and line type denote season.
**Figure S16:** Principal coordinates analysis with 95% confidence ellipses of samples collected from *Heteronotia binoei*. Colors denote sampling site. Point shape and line type (bottom panels) denote season.

**Table S12:** emi470172‐sup‐0002‐TableS12.xlsx.

## Data Availability

Bacterial amplicon sequence data are available on NCBI's Sequence Read Archive (BioProject accession: PRJNA1151157). Environmental data included in the principal components analysis are available in Supplemental Table S12.
