# Supplementary material for: Unravelling Intrinsic and Extrinsic Factors Shaping the Rich Communities on Lizard Skin
Source: Environ Microbiol Rep. 2025 Aug 6;17(4):e70172. doi: 10.1111/1758-2229.70172 (PMC12328065; doi:10.1111/1758-2229.70172)
Supplement: Supplementary file 1 — Table S1: Five sampling sites in the Northern Territory, Australia and their weather station data. Distance ranges are included for the three sites with local subsites. Table S2: Sample sizes from local subsites per sampling location. At Litchfield National Park, Tolmer Falls is 13+ km from the other local sites, which are within 2.75 km of each other. At Timber Creek, all local sites are within 2.8 km of each other. At Kidman Springs, the quarry site is approx. 6.25 km from the dump, which is 1 km from the station. Samples from Charles Darwin University and Katherine are excluded here, because at each, the samples were collected within ~0.5 km of each other. Table S3: Core ASVs on the skin of groups of geckos (species or site) at 80% prevalence cut‐off. Asterisks at the beginning of ASV codes indicate ASVs present in the cores of multiple groups. Table S4: Core genera on the skin of gecko species at 80% prevalence cut‐off. Table S5: Results of pairwise PERMANOVA by species for samples from Litchfield National Park. Table S6: Results of pairwise PERMANOVA by species for samples from Katherine. Table S7: Results of pairwise PERMANOVA by species for samples from Kidman Springs. Table S8: Results of pairwise PERMANOVA by site for samples from Gehyra nana . Table S9: Results of analyses of beta dispersion with permdisp by sampling site. Each variable was analyzed separately. Differences in beta dispersion were only assessed for variables significant in PERMANOVA. Bold denotes significance. Table S10: Results of analyses of beta dispersion with permdisp by host species (Gehyra spp. and Heteronotia binoei). Each variable was analysed separately. Differences in beta dispersion were only assessed for variables significant in PERMANOVA. Bold denotes significance. Table S11: p values from pairwise PERMANOVA for unweighted UniFrac in Gehyra nana skin bacteria. Figure S1: Multiple environmental variables were collected for each sampling location and date, representing recent an [file EMI4-17-e70172-s002.pdf]

Supplemental Materials

Unravelling intrinsic and extrinsic factors shaping the rich communities on lizard skin

Chava L. Weitzman[1], Kimberley Day[1], Karen Gibb[1], Gregory P. Brown[2], Angga Rachmansah[1], Keith Christian[1]

[1] Research Institute for the Environment and Livelihoods, CDU, Casuarina, NT, Australia

[2] School of Natural Sciences, Macquarie University, Sydney, NSW, Australia

Corresponding author: Chava Weitzman, chava.weitzman2@cdu.edu.au, Research Institute for the Environment and Livelihoods, CDU, Casuarina, NT, Australia

Environmental Microbiology Reports, Article DOI: 10.1111/1758-2229.70172

Table S1 Five sampling sites in the Northern Territory, Australia and their weather station data. Distance ranges are included for the three sites with local subsites.

**Site**	**Latitude**	**Longitude**	**Temperature Weather Station Number**	**Temperature Weather Station Distance (km)**	**Rainfall Weather Station Number**	**Rainfall Weather Station Distance (km)**
Charles Darwin University	-12.3691	130.8671	14015	6	14285	<3
Katherine	-14.3998	132.1420	14932	29	14927	3
Kidman Springs	-16.1291	130.9360	14825	31–32	14847	<0.5–7
Litchfield National Park	-13.1595	130.7629	14272	24–38	14279	11–14
Timber Creek	-15.6378	130.4543	14808	24–26	14660	3–5

Table S2 Sample sizes from local subsites per sampling location. At Litchfield National Park, Tolmer Falls is 13+ km from the other local sites, which are within 2.75 km of each other. At Timber Creek, all local sites are within 2.8 km of each other. At Kidman Springs, the quarry site is approx. 6.25 km from the dump, which is 1 km from the station. Samples from Charles Darwin University and Katherine are excluded here, because at each, the samples were collected within ~0.5 km of each other.

**Litchfield National Park**	*G. australis*	*G. lapistola*	*G. nana*	*G. paranana*	*H. binoei*
Buley Rockhole					4
Intersection		10	3	12	
Nana Hill			4		3
Tolmer Falls	14	2	7	2	7

**Timber Creek**	*G. gemina*	*G. koira*	*G. nana*	*H. binoei*
Dump	3		1	7
Cemetery	6			
Hill		1	5	
Mid-road	3		2	
Lookout	2	12	6	

**Kidman Springs**	*G. gemina*	*G. koira*	*G. nana*	*H. binoei*
Station			4	
Dump	12		12	10
Quarry	2	15		

Table S3 Core ASVs on the skin of groups of geckos (species or site) at 80% prevalence cut-off. Asterisks at the beginning of ASV codes indicate ASVs present in the cores of multiple groups.

**Gecko Group**	**ASV Taxon**	**ASV Code**	**Median Relative Abundance (%)**	**98% Relative Abundance (%)**
G. australis (n = 30)	*Flavobacterium*	*1aa7ac9dc8420d7ad71b153f1a417f71	0.8	6.1
	Methylobacterium-Methylorubrum	*6152e64914f4cc0e78c131533f217165	0.3	10.5
G. gemina (n = 28)	*Enhydrobacter*	*0d981b8b7342dbd090a2cea60adae3b8	0.6	6.1
	Flavobacterium	*1aa7ac9dc8420d7ad71b153f1a417f71	0.7	4.5
	Methylobacterium-Methylorubrum	*6152e64914f4cc0e78c131533f217165	0.2	5.8
G. koira (n = 28)		NA		
G. lapistola (n = 12)	*Enhydrobacter*	*0d981b8b7342dbd090a2cea60adae3b8	0.1	5.4
	Flavobacterium	*1aa7ac9dc8420d7ad71b153f1a417f71	0.2	4.9
	Staphylococcus	*ca497db8096dc55eb7e2b150b2d3bb8d	0.3	5.8
	Micrococcus	d2cd728895838a74dbafe2874e046ab3	0.3	1.6
G. nana (n = 58)		NA		
G. paranana (n = 14)	*Enhydrobacter*	*0d981b8b7342dbd090a2cea60adae3b8	0.5	6.8
	Flavobacterium	*1aa7ac9dc8420d7ad71b153f1a417f71	1.0	5.6
	Methylobacterium-Methylorubrum	*6152e64914f4cc0e78c131533f217165	0.4	1.1
	Gammaproteobacteria	992b6dc32295d9c78f4023d01d1eeb41	1.0	9.3
	Bacteroidetes bacterium	c459e95b26174648f29471d887e22d1b	9.4	18.0
	Staphylococcus	*ca497db8096dc55eb7e2b150b2d3bb8d	0.6	7.7
	Bacteria	fc9897aa27ac7a7b9c2ab39f32820351	1.8	10.2
H. binoei (n = 60)	*Flavobacterium*	*1aa7ac9dc8420d7ad71b153f1a417f71	0.2	2.9
Charles Darwin University (n = 14)	*Flavobacterium*	1aa7ac9dc8420d7ad71b153f1a417f71	0.7	2.5
	Actinomycetospora	3f06b59f06dcea266fa517b1e42d337f	1.3	9.1
	Bacteroidetes bacterium	ec373217d0ad55b869c78d72364e0091	1.8	13.9
Litchfield National Park (n = 68)	*Flavobacterium*	1aa7ac9dc8420d7ad71b153f1a417f71	0.6	6.4
Katherine (n = 45)		NA		
Timber Creek (n = 48)	*Flavobacterium*	1aa7ac9dc8420d7ad71b153f1a417f71	0.3	2.4
Kidman Springs (n = 55)	*Enhydrobacter*	0d981b8b7342dbd090a2cea60adae3b8	0.2	5.4
	Flavobacterium	1aa7ac9dc8420d7ad71b153f1a417f71	0.2	3.5
	Methylobacterium-Methylorubrum	6152e64914f4cc0e78c131533f217165	0.3	6.3
	Staphylococcus	ca497db8096dc55eb7e2b150b2d3bb8d	0.2	6.5

Table S4 Core genera on the skin of gecko species at 80% prevalence cut-off.

Gecko Group	Genus	Median Relative Abundance (%)	98% Relative Abundance (%)
G. australis	*Actinomycetospora*	1.4	12.7
	Streptomyces	0.3	10.4
	Flavobacterium	0.8	6.2
	Methylobacterium-Methylorubrum	1.1	16.7
	Sphingomonas	1.2	13.2
	Pseudomonas	0.3	3.6
G. gemina	*Actinomycetospora*	0.5	19.5
	Streptomyces	0.5	7.6
	Bacteroides	4.9	28.8
	Parabacteroides	2.1	19.7
	Flavobacterium	0.7	4.5
	Staphylococcus	0.5	6.4
	Lachnoclostridium	0.7	11.7
	Methylobacterium-Methylorubrum	0.8	13.7
	Sphingomonas	1.8	9.3
	Enhydrobacter	0.7	6.1
G. koira	*Pseudonocardia*	0.2	3.0
	Streptomyces	0.7	8.2
	Bacteroides	15.6	33.3
	Parabacteroides	3.9	21.3
	Lachnoclostridium	1.5	9.0
	Methylobacterium-Methylorubrum	0.4	18.7
	Sphingomonas	0.3	11.4
G. lapistola	*Bryocella*	0.4	3.9
	Mycobacterium	0.2	1.7
	Micrococcus	0.3	1.6
	Conexibacter	0.2	2.5
	Bacteroides	15.3	39.1
	Odoribacter	0.2	2.1
	Flavobacterium	0.2	6.0
	Staphylococcus	0.3	5.9
	Methylobacterium-Methylorubrum	0.2	2.3
	Sphingomonas	0.4	4.2
	Acinetobacter	0.1	5.5
	Enhydrobacter	0.1	6.1
G. nana	*NA*		
G. paranana	*Bryocella*	0.4	7.5
	Corynebacterium	0.3	6.9
	Mycobacterium	0.3	2.0
	Actinomycetospora	0.3	2.3
	Pseudonocardia	0.3	4.2
	Conexibacter	0.4	2.8
	Flavobacterium	1.0	5.6
	Bacillus	0.1	1.4
	Staphylococcus	1.0	7.7
	1174-901-12	0.3	2.3
	Methylobacterium-Methylorubrum	0.5	2.3
	Sphingomonas	0.5	3.8
	Acinetobacter	0.3	2.9
	Enhydrobacter	0.5	6.8
	Pseudomonas	0.3	2.1

[Table S4 cont.]

Gecko Group	Genus	Median %	98% Abund.
H. binoei	*Pseudonocardia*	0.3	7.3
	Streptomyces	0.8	8.6
	Flavobacterium	0.2	3.0
	Methylobacterium-Methylorubrum	0.4	8.5
	Sphingomonas	0.6	7.1
	Pseudomonas	0.1	1.0

Table S5 Results of pairwise PERMANOVA by species for samples from Litchfield National Park. * = significance in unweighted UniFrac distance. * = significance in weighted UniFrac distance.

Litchfield National Park	*G. lapistola*	*G. nana*	*G. paranana*	*H. binoei*
G. australis	ns	**	**	**
G. lapistola		*	**	*
G. nana			*	*
G. paranana				*

Table S6 Results of pairwise PERMANOVA by species for samples from Katherine. * = significance in unweighted UniFrac distance. * = significance in weighted UniFrac distance.

Katherine	*G. nana*	*H. binoei*
G. australis	**	**
G. nana		*

Table S7 Results of pairwise PERMANOVA by species for samples from Kidman Springs. * = significance in unweighted UniFrac distance. * = significance in weighted UniFrac distance.

Katherine	*G. koira*	*G. nana*	*H. binoei*
G. gemina	**	**	**
G. koira		**	**
G. nana			**

Table S8 Results of pairwise PERMANOVA by site for samples from *Gehyra nana*. * = significance in unweighted UniFrac distance. There were no significant differences between sites in weighted UniFrac distance.

G. nana	Katherine	Timber Creek	Kidman Springs
Litchfield National Park	*	*	*
Katherine			*
Timber Creek			

Table S9 Results of analyses of beta dispersion with permdisp by sampling site. Each variable was analyzed separately. Differences in beta dispersion were only assessed for variables significant in PERMANOVA. **Bold** denotes significance.

Site	Variable	Unweighted UniFrac		Weighted UniFrac	
		F	P	F	P
Timber Creek	Species	0.146	0.9		
Kidman Springs	**Species**	0.482	0.7	3.788	**0.02**
	Season	0.786	0.4		
Katherine	Species	0.022	1.0	0.914	0.3
	**Season**	1.420	0.2	4.877	**0.02***
Litchfield	**Species**	1.265	0.2	3.080	**0.009**
	Season	1.672	0.2	0.349	0.6
Charles Darwin Uni	Season	0.631	0.4	1.230	0.2

* In Katherine, wet season samples were more similar than dry season samples.

Table S10 Results of analyses of beta dispersion with permdisp by host species (*Gehyra* spp. and *Heteronotia binoei*). Each variable was analyzed separately. Differences in beta dispersion were only assessed for variables significant in PERMANOVA. **Bold** denotes significance.

Species	Variable	Unweighted UniFrac		Weighted UniFrac	
		F	P	F	P
G. australis	Season	0.047	0.8	0.431	0.4
G. gemina	Site	0.257	0.6	0.115	0.7
	Season	0.013	0.9		
G. koira	Site	0.363	0.5		
	Season	4.174	0.053	1.387	0.2
G. lapistola	Season	1.050	0.3	0.556	0.4
G. nana	Site	1.567	0.2	1.748	0.2
	**Season**	3.532	0.056	8.548	**0.009***
G. paranana	**Season**	2.784	0.06	12.847	**0.003**#
H. binoei	Site	0.674	0.7	0.932	0.3
	Season	0.696	0.4	0.582	0.4

*For *G. nana*, wet season samples were more similar than dry season samples.
#For *G. paranana*, dry season samples were more similar than wet season samples.

Table S11 P-values from pairwise PERMANOVA for unweighted UniFrac in *Gehyra nana* skin bacteria. Green = significant differences between seasons in a site. Red = significant differences between sites within a season. Grey = nonfocal comparison. "x" = not significant (p > 0.05). KAT = Katherine. KS = Kidman Springs. LNP = Litchfield National Park. TC = Timber Creek.

	KAT wet	KS dry	KS wet	LNP dry	LNP wet	TC dry	TC wet
KAT dry	0.045	x	0.006	0.008	x	x	x
KAT wet		0.008	0.006	0.006	x	x	x
KS dry			x	0.008	0.03	x	x
KS wet				0.006	0.03	x	x
LNP dry					0.008	0.006	0.008
LNP wet						x	x
TC dry							x

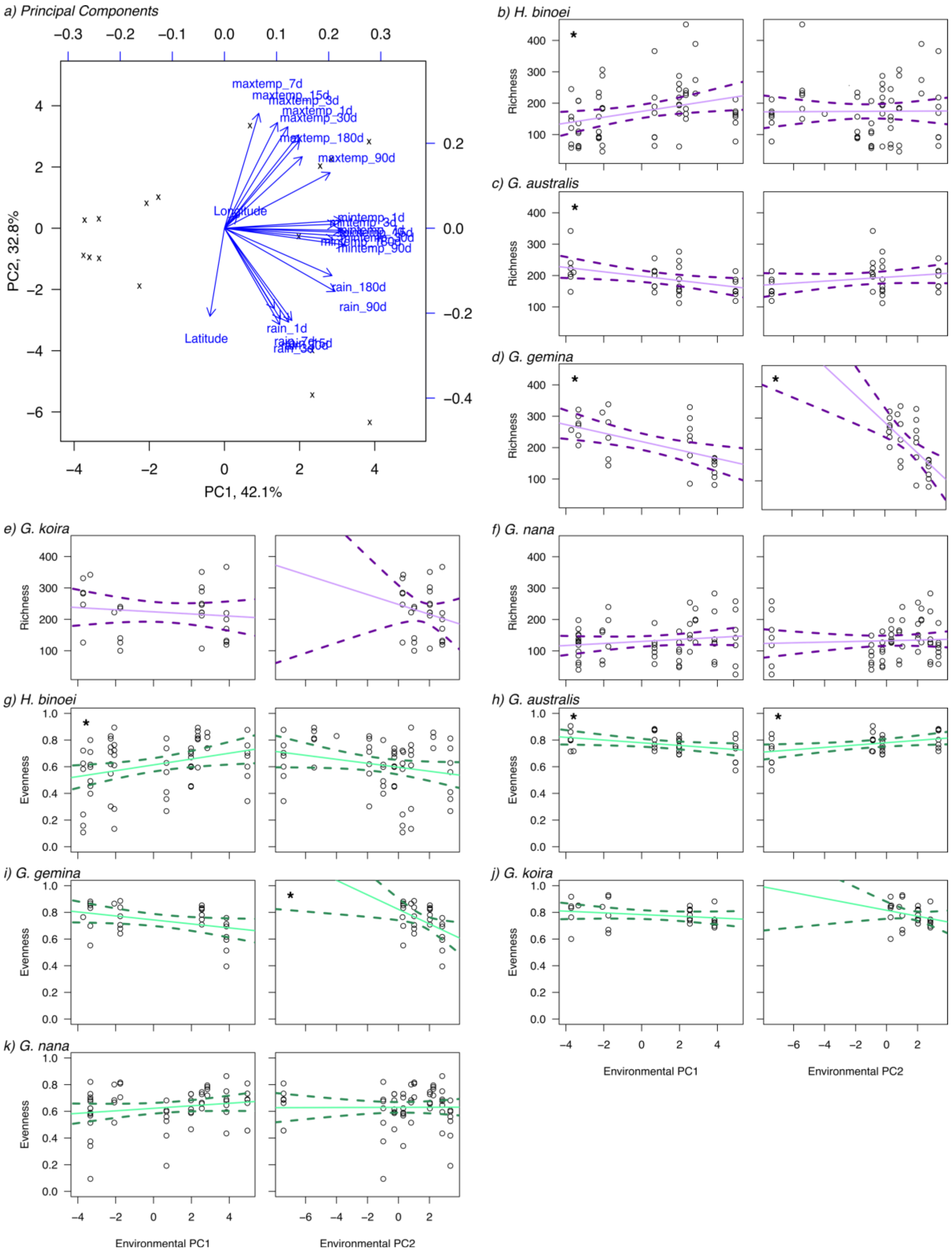

Figure S1 Multiple environmental variables were collected for each sampling location and date, representing recent and long-term (up to 180 days) weather experienced by the host geckos and their skin microbiomes. These environmental variables were collapsed using a principal components analysis, for which the first two axes (a) accounted for 75% of the variation. Environmental PC1 and PC2 were analysed as predictor variables for richness and evenness on each gecko species sampled from more than one location. (b-f) Raw values and

[Figure S1 cont.] confidence intervals of the relationships between bacterial richness and PC1 (left panels) or PC2 (right panels) for each gecko species. (g-k) Raw values and confidence intervals of the relationships between Pielou's evenness and PC1 (left panels) or PC2 (right panels) for each gecko species. Asterisks denote significant associations.

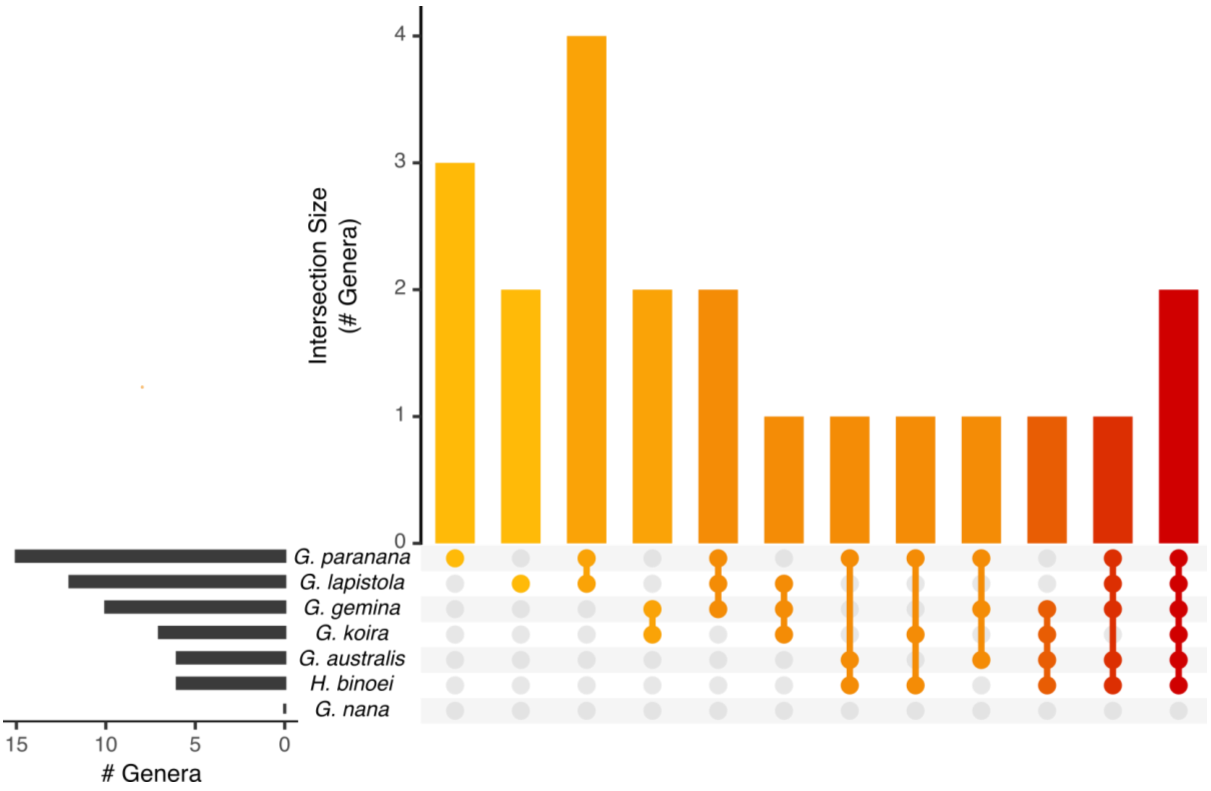

Figure S2 UpSet plot depicting unique and overlapping core genera in *Gehyra* spp. and *Heteronotia binoei*. Warmer colors identify genera in core communities shared by more species.

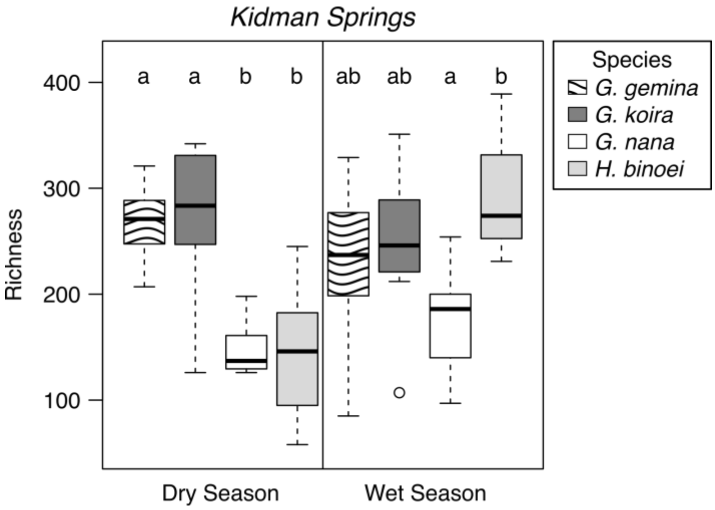

Figure S3 Bacterial richness among the gecko species in the two seasons at the Kidman Springs site, the only site which had a significant interaction between species and season. Letters above boxplots indicate post-hoc differences in richness between species within each season separately to visualize the many differences in the dry season not found in the wet. Post-hoc tests detected greater richness on *H. binoei* in the wet season versus the dry season.

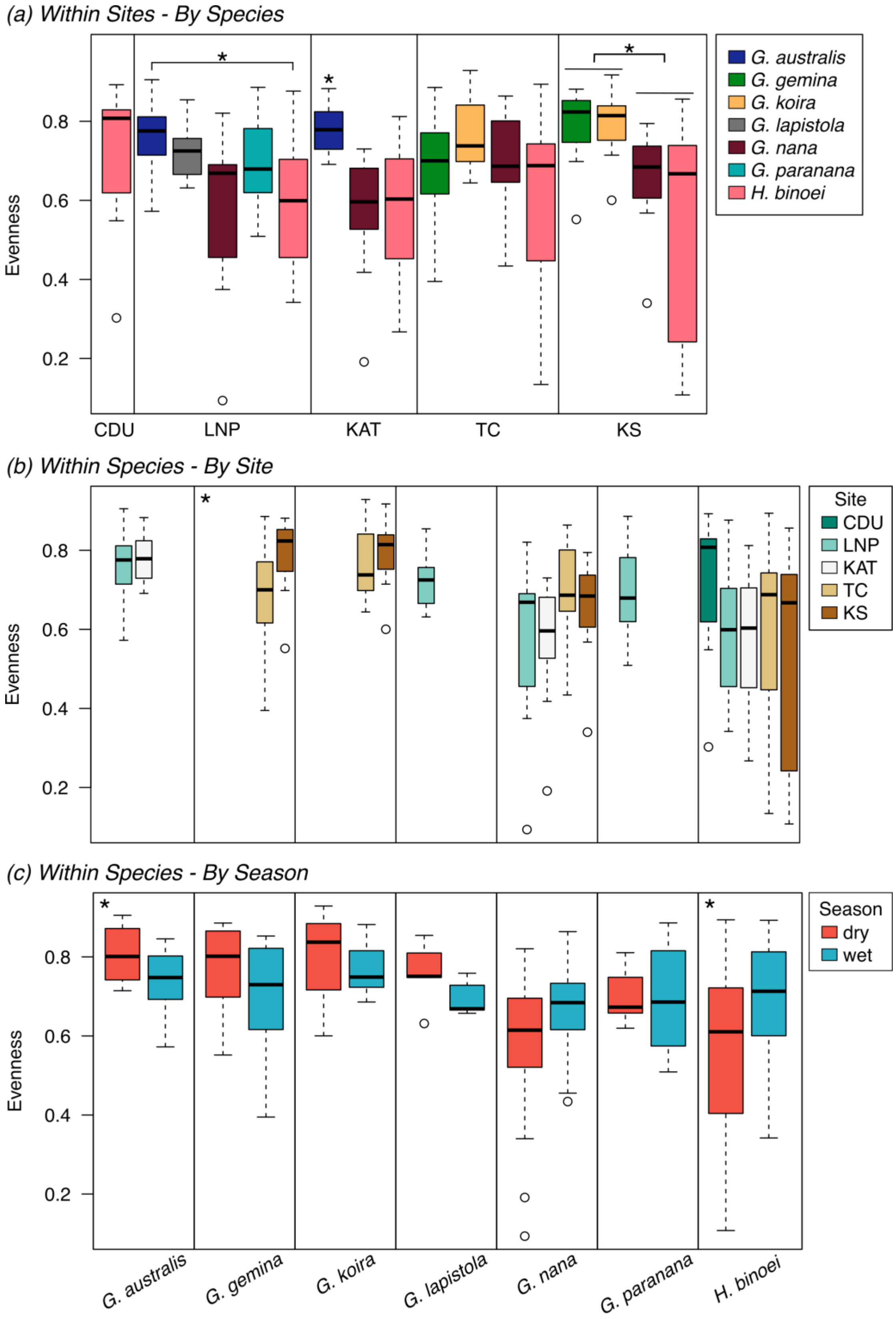

Figure S4 Pielou's evenness in skin bacterial communities on *Gehyra* spp. and *Heteronotia binoei* geckos among a) species per site, b) sites per species, and c) season per species. Sites arranged north to south, corresponding with decreasing annual precipitation. Asterisks denote significant differences.

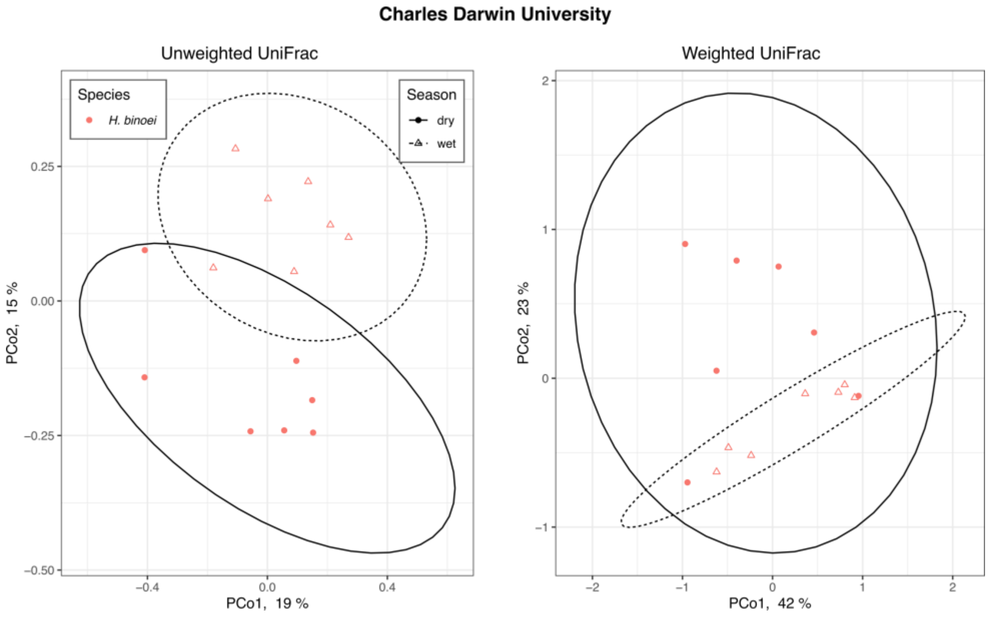

Figure S5 Principal coordinates analysis with 95% confidence ellipses of samples collected from geckos (*Heteronotia binoei*) at Charles Darwin University. Point shape and line type denote season.

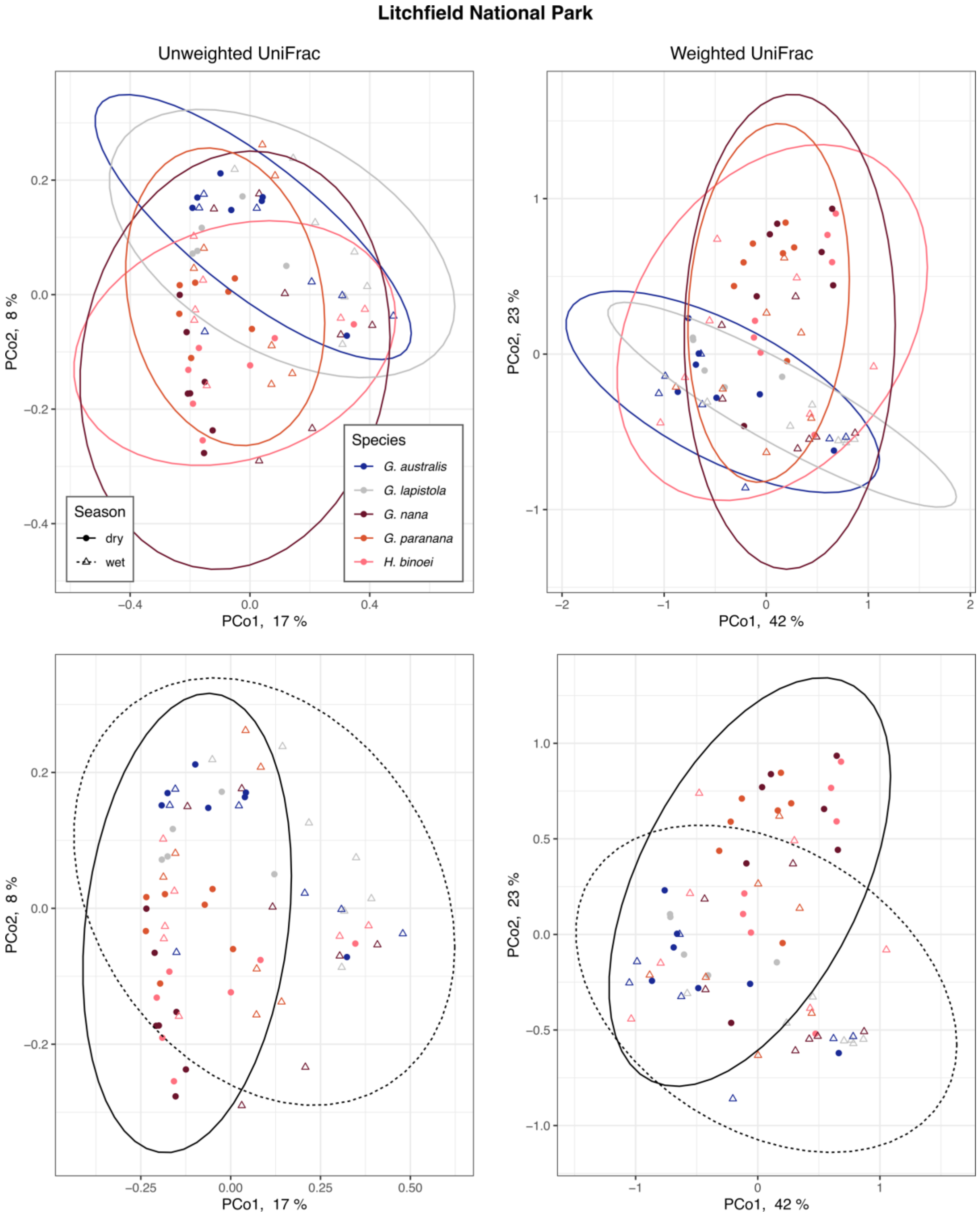

Figure S6 Principal coordinates analysis with 95% confidence ellipses of samples collected from geckos at Litchfield National Park. Color denote species. Point shape and line type (bottom panels) denote season.

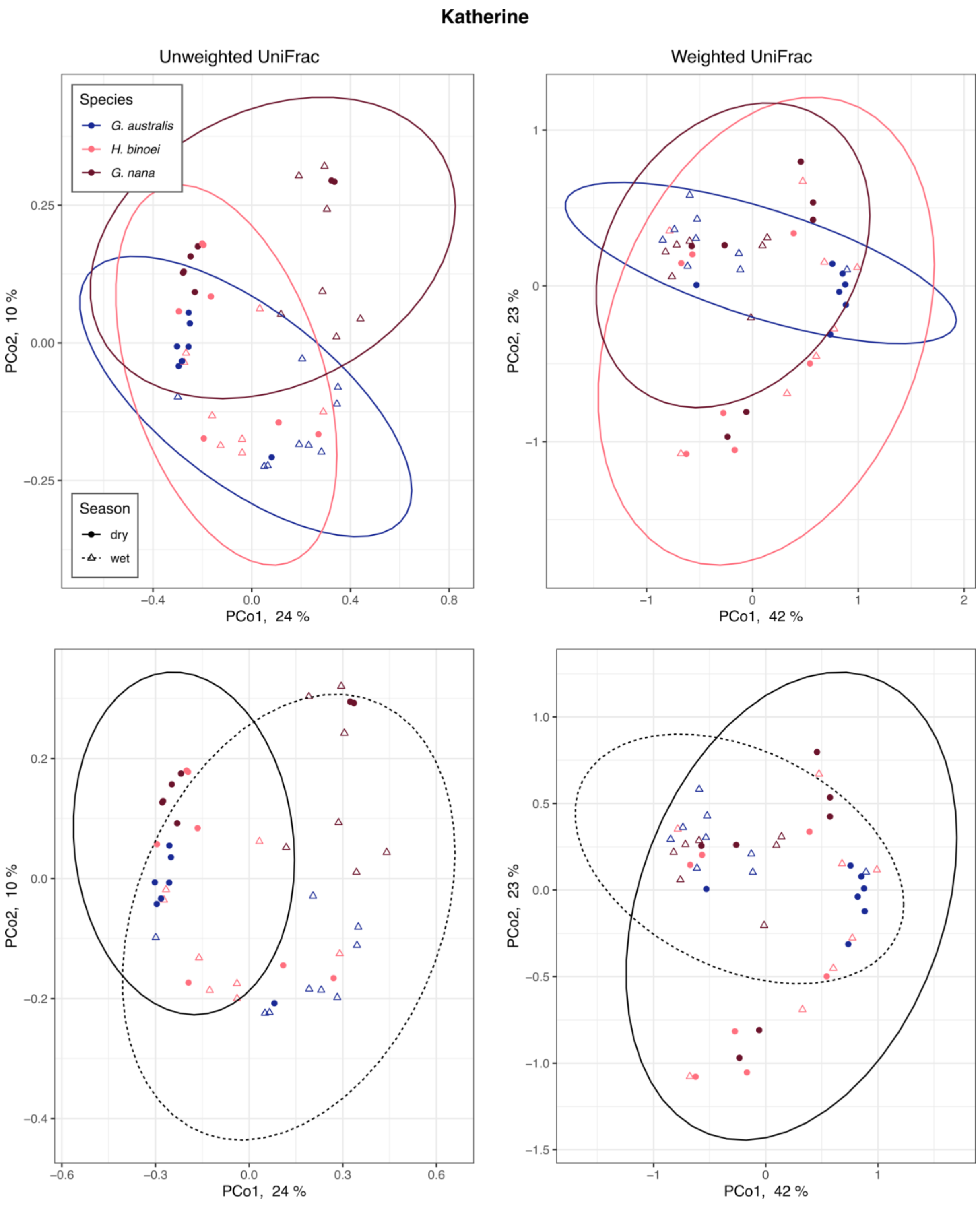

Figure S7 Principal coordinates analysis with 95% confidence ellipses of samples collected from geckos at Katherine. Colors denote species. Point shape and line type (bottom panels) denote season.

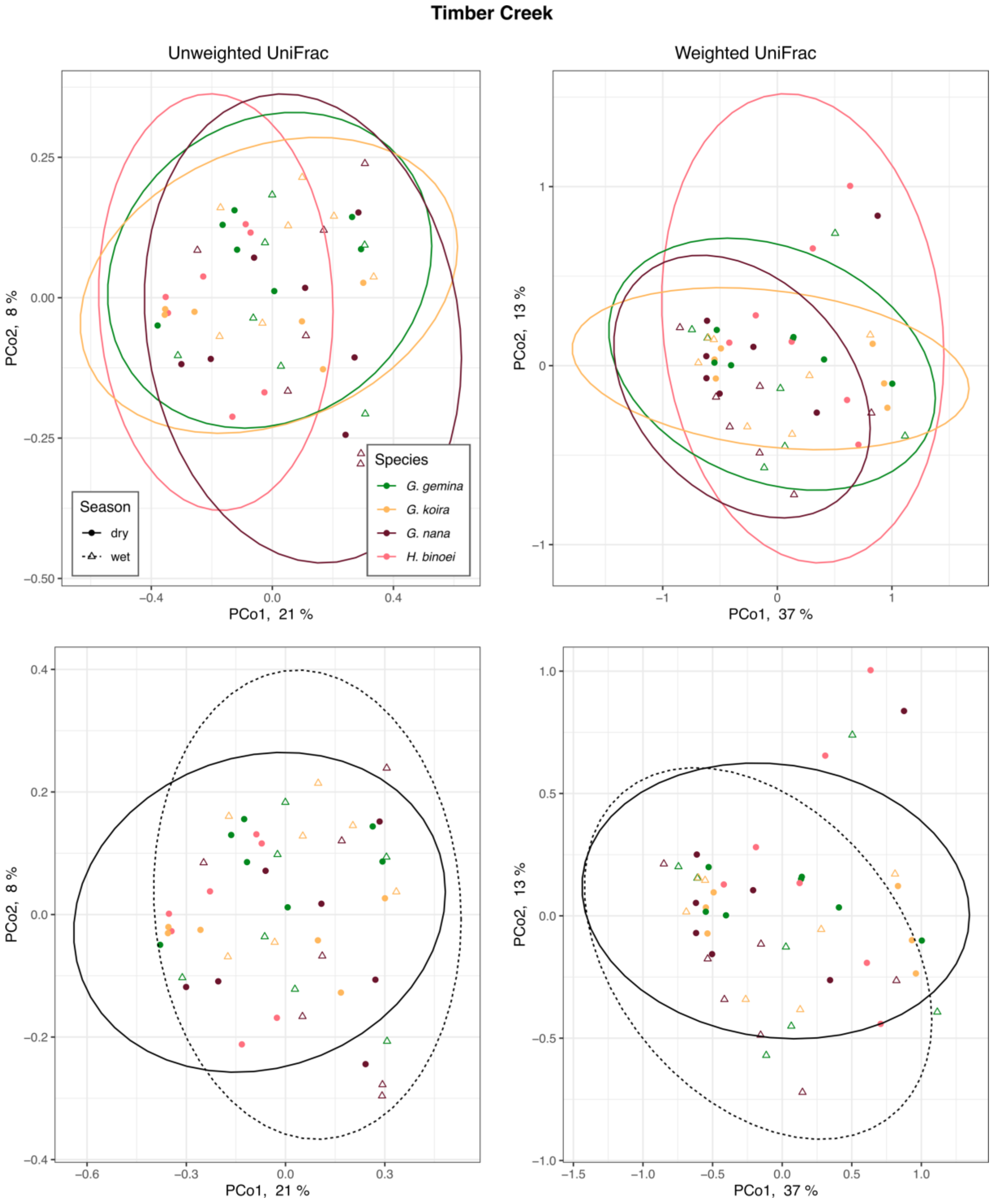

Figure S8 Principal coordinates analysis with 95% confidence ellipses of samples collected from geckos at Timber Creek. Colors denote species. Point shape and line type (bottom panels) denote season.

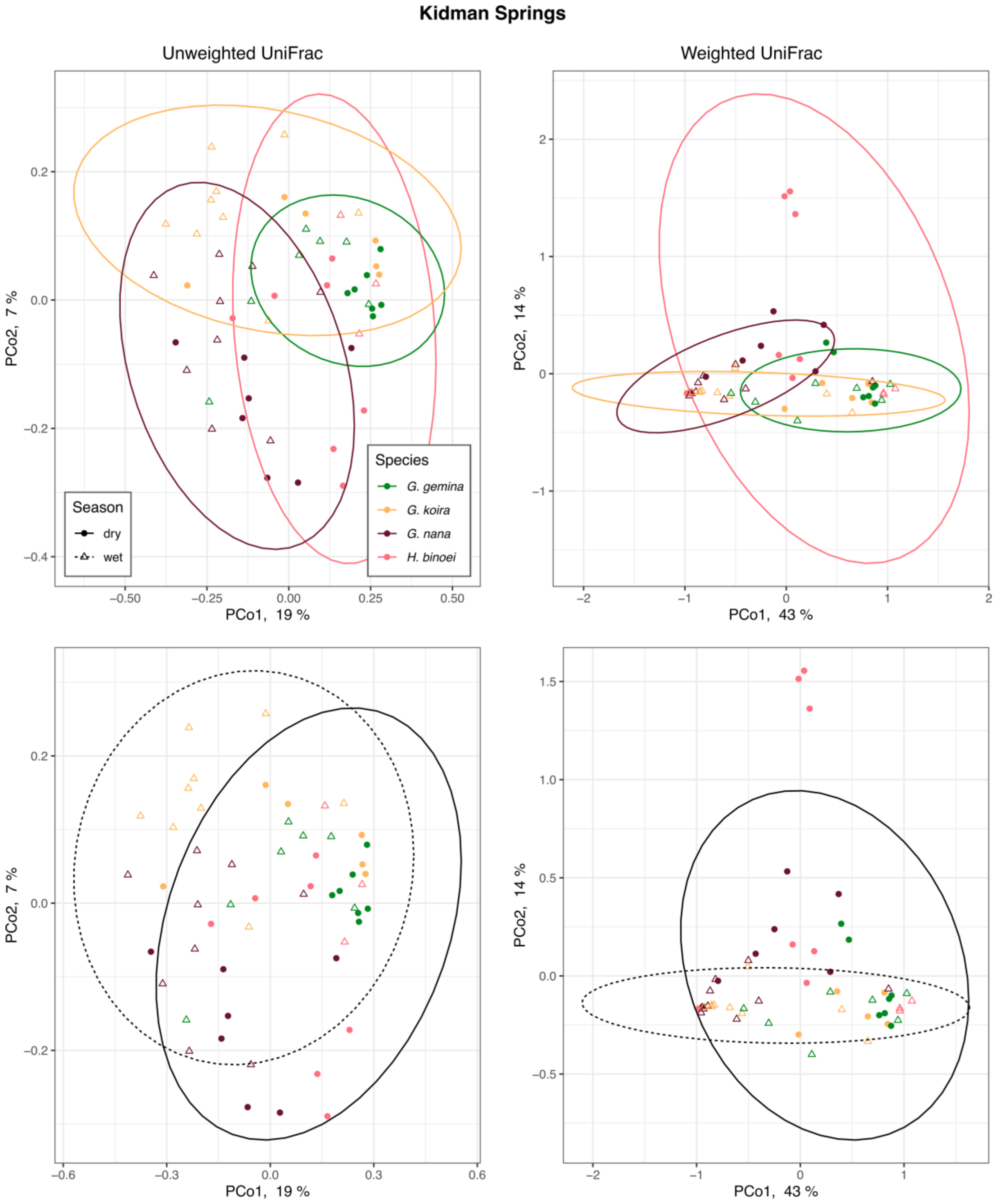

Figure S9 Principal coordinates analysis with 95% confidence ellipses of samples collected from geckos at Kidman Springs. Colors denote species. Point shape and line type (bottom panels) denote season.

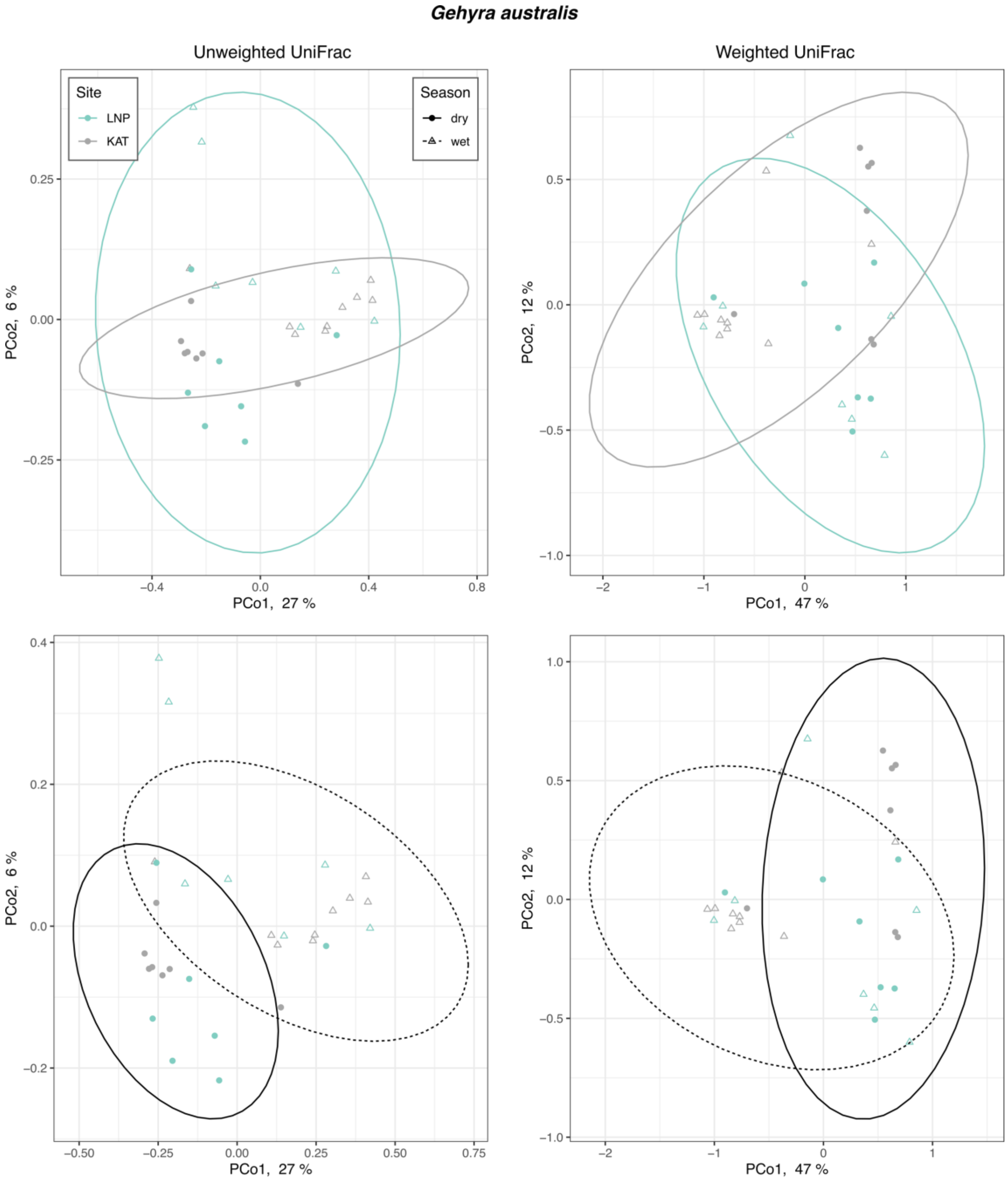

Figure S10 Principal coordinates analysis with 95% confidence ellipses of samples collected from *Gehyra australis*. Colors denote sampling site. Point shape and line type (bottom panels) denote season.

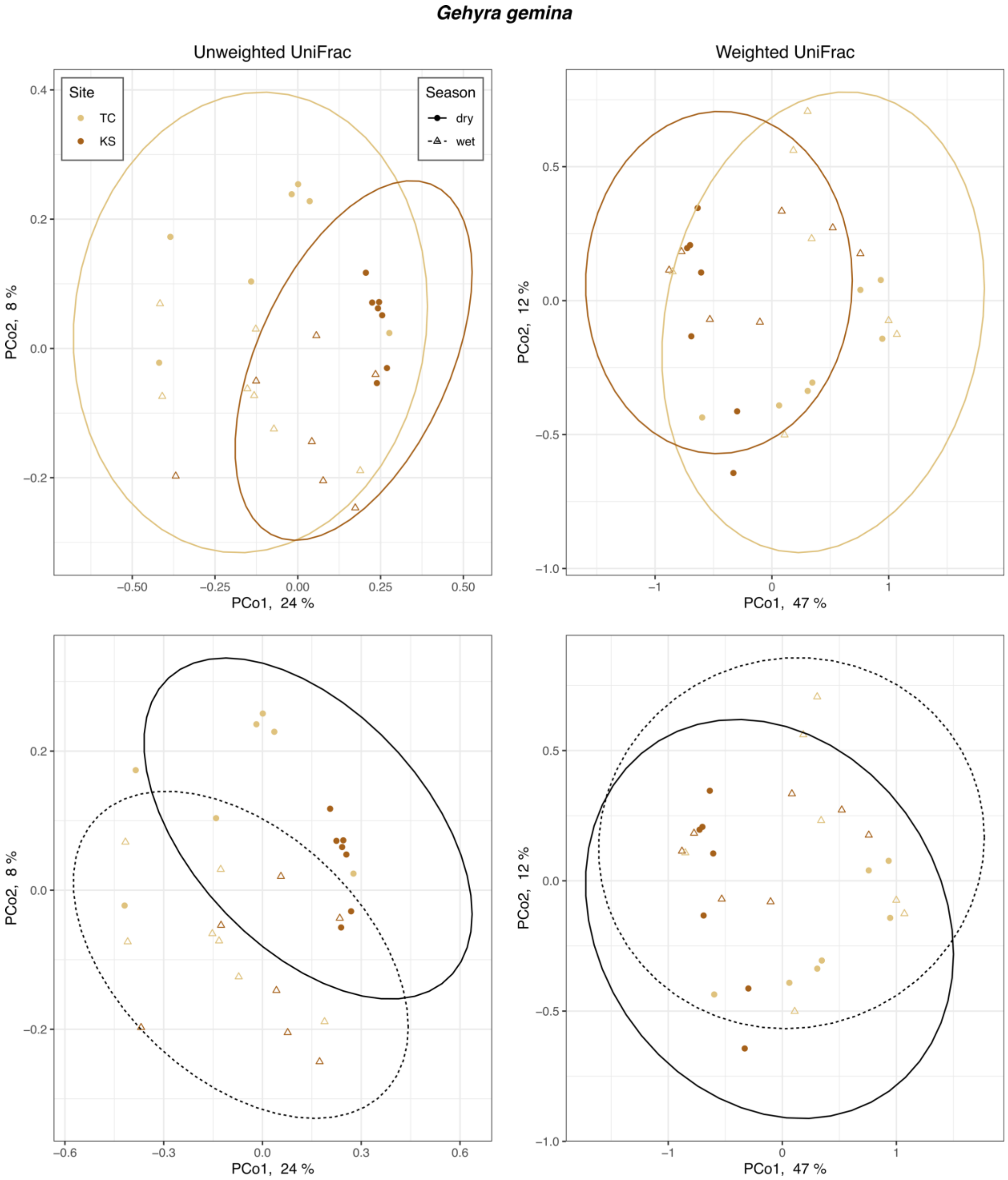

Figure S11 Principal coordinates analysis with 95% confidence ellipses of samples collected from *Gehyra gemina*. Colors denote sampling site. Point shape and line type (bottom panels) denote season.

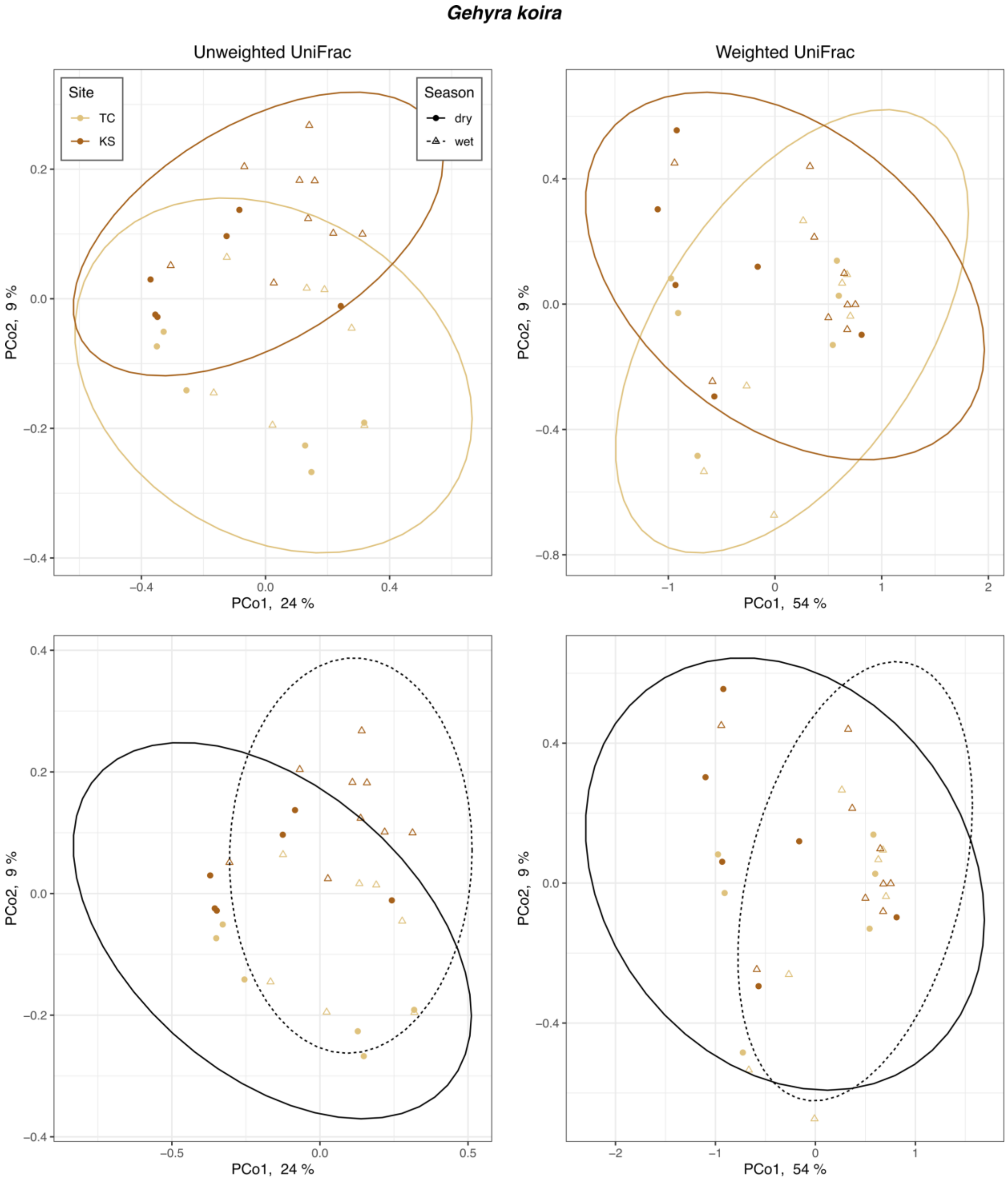

Figure S12 Principal coordinates analysis with 95% confidence ellipses of samples collected from *Gehyra koira*. Colors denote sampling site. Point shape and line type (bottom panels) denote season.

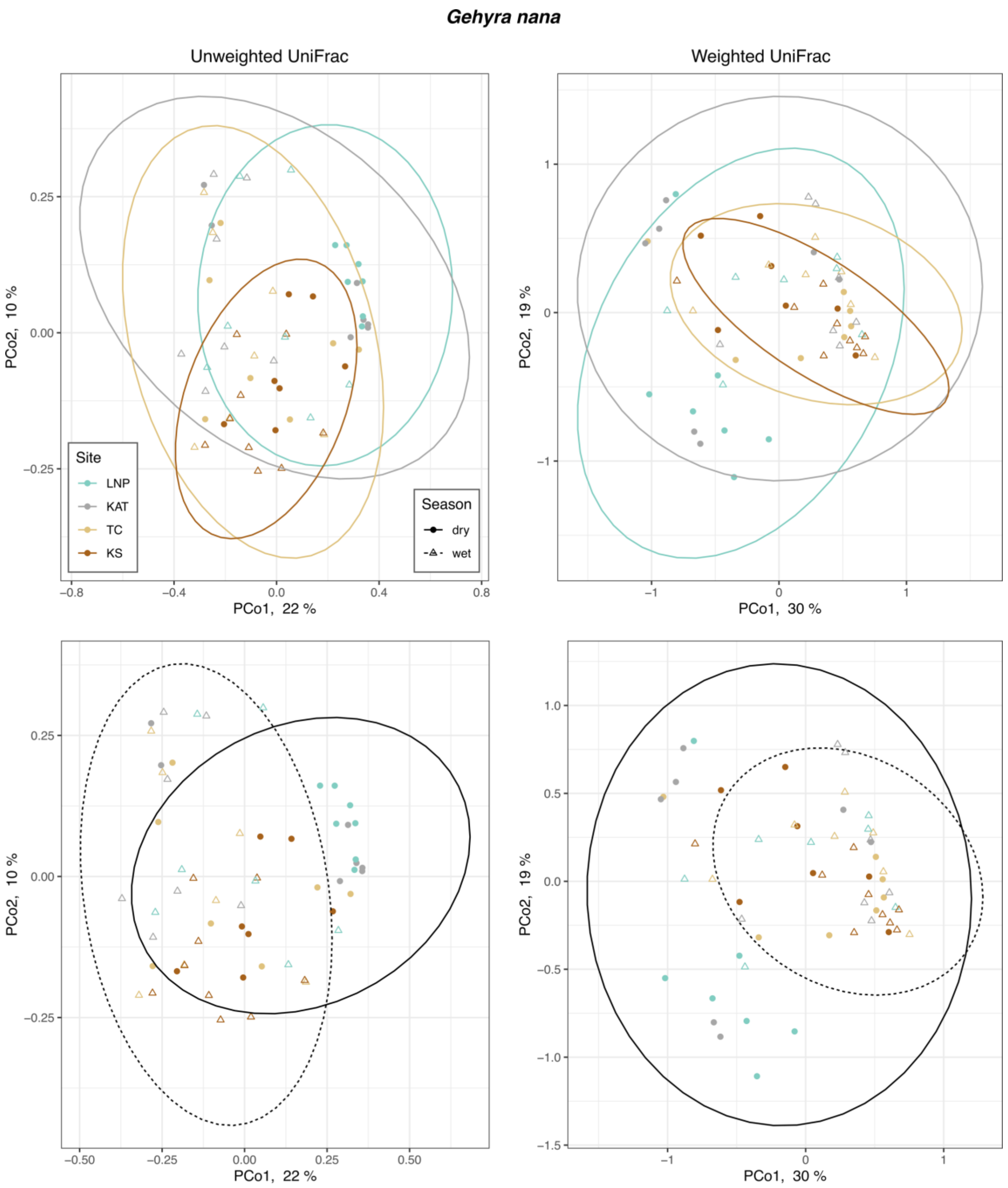

Figure S13 Principal coordinates analysis with 95% confidence ellipses of samples collected from *Gehyra nana*. Colors denote sampling site. Point shape and line type (bottom panels) denote season.

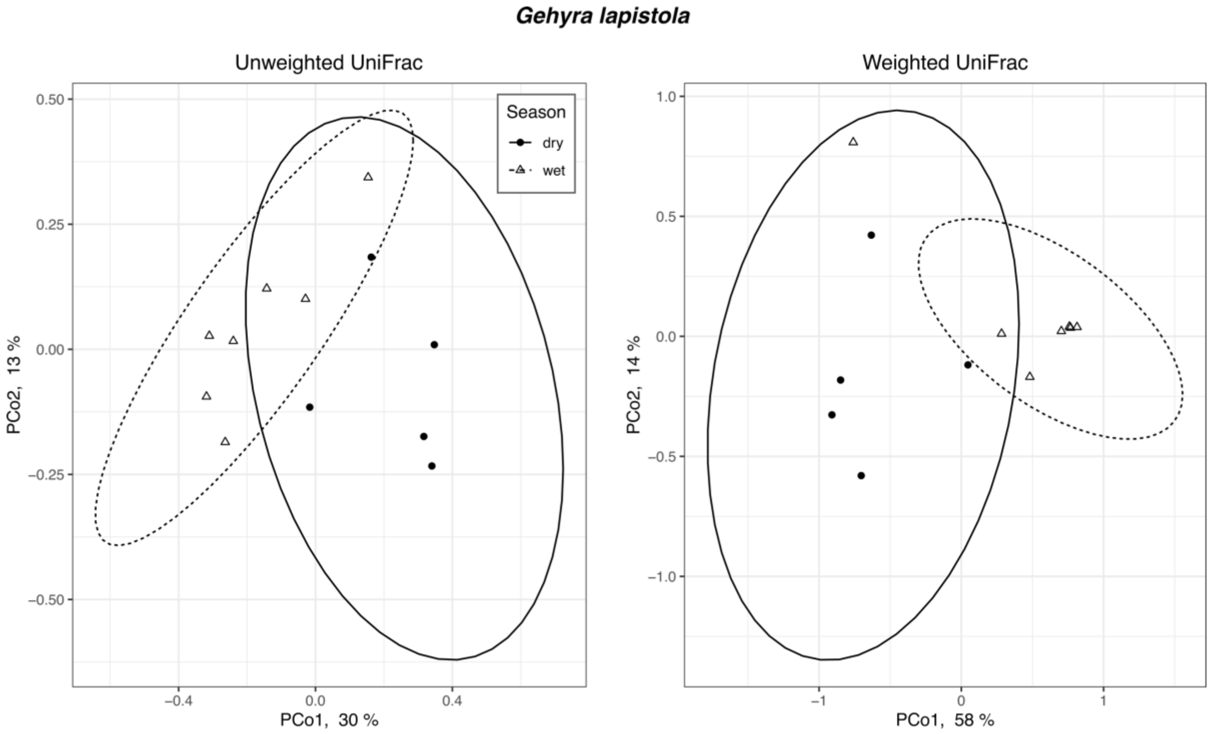

Figure S14 Principal coordinates analysis with 95% confidence ellipses of samples collected from *Gehyra lapistola* at Litchfield National Park. Point shape and line type denote season.

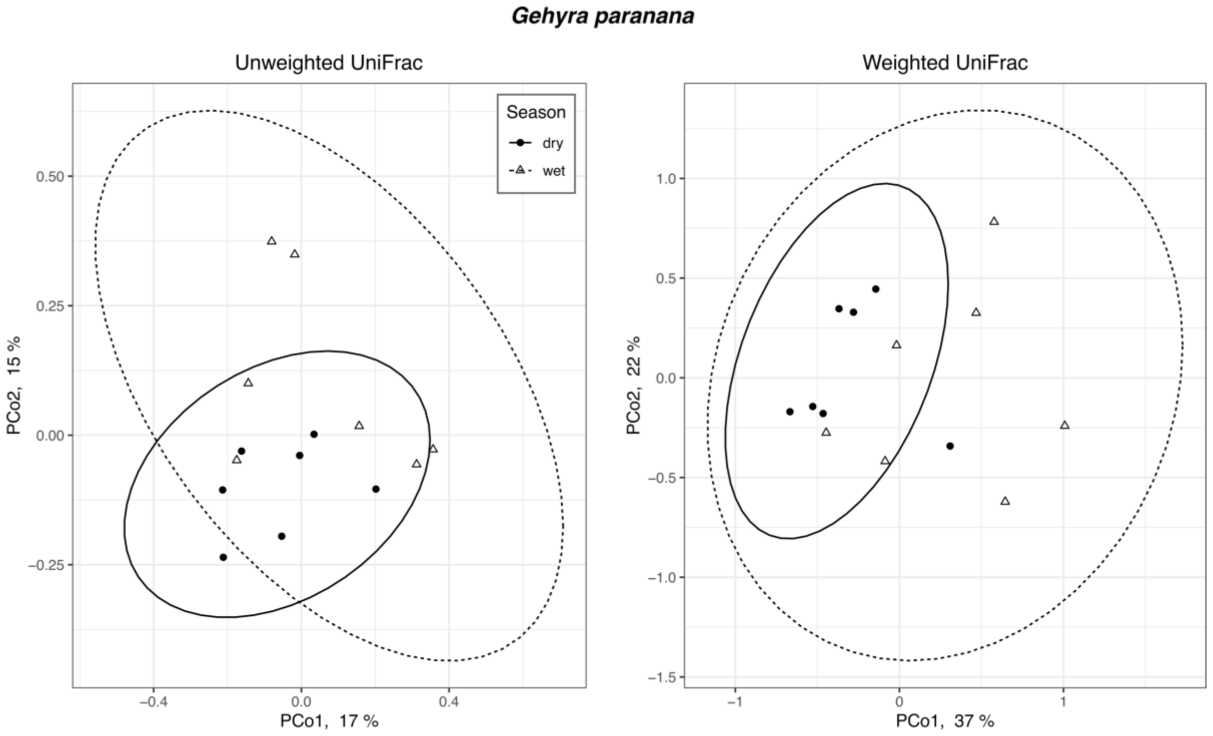

Figure S15 Principal coordinates analysis with 95% confidence ellipses of samples collected from *Gehyra paranana* at Litchfield National Park. Point shape and line type denote season.

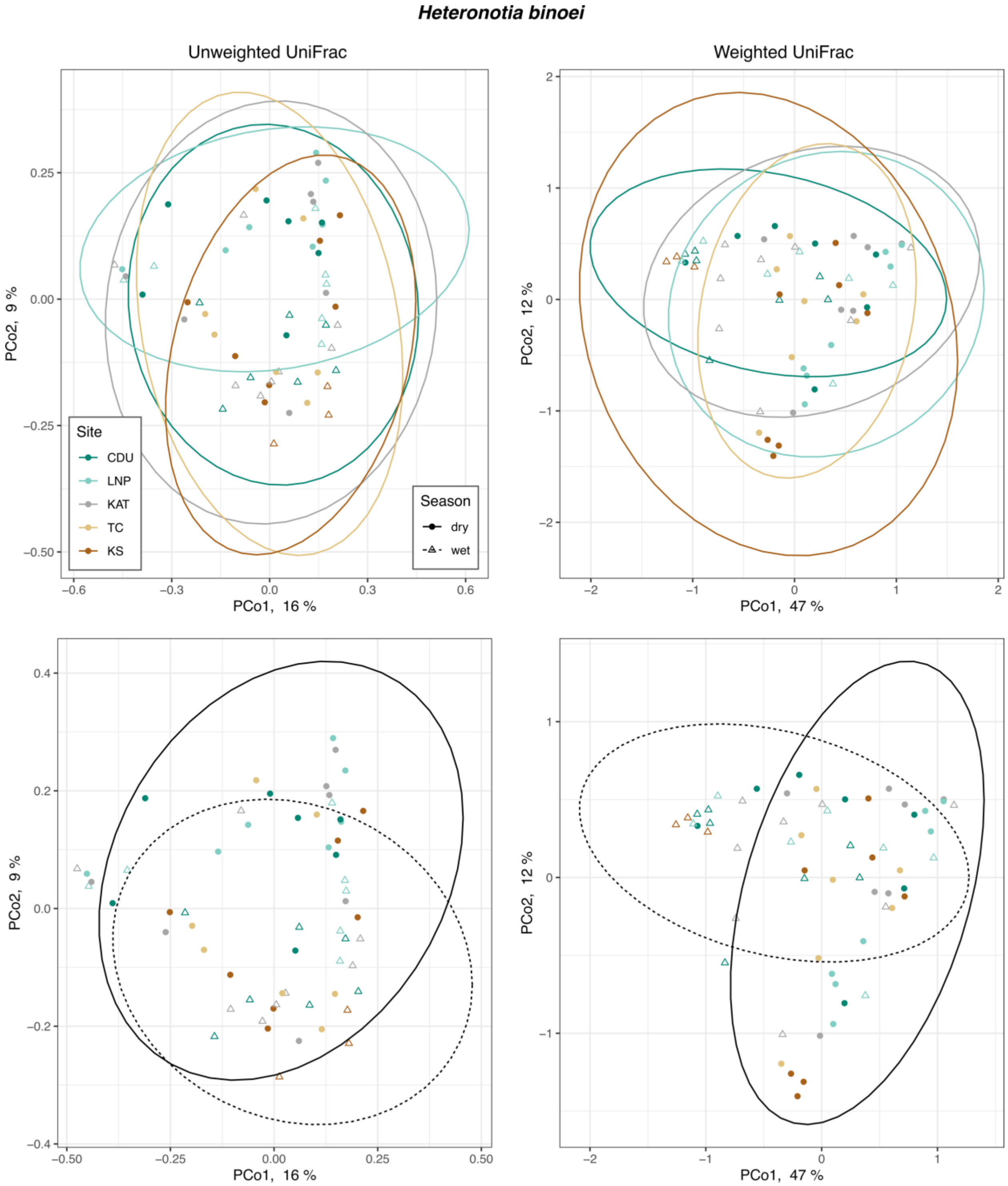

Figure S16 Principal coordinates analysis with 95% confidence ellipses of samples collected from *Heteronotia binoei*. Colors denote sampling site. Point shape and line type (bottom panels) denote season.
